# Retrieval of Soybean SPAD at Different Vertical Canopy Scales Based on Fractional-Order Derivatives and Spectral Indices

**DOI:** 10.3390/plants15192998

**Published:** 2026-09-30

**Authors:** Jinqi Wu, Wangyang Li, Shiqi Liu, Wenting Hou, Shuai Li, Zijun Tang, Ruiqi Du, Junsheng Lu, Youzhen Xiang

**Affiliations:** 1 College of Water Resources and Architectural Engineering, Northwest A&F University, Yangling 712100, China; 2025056093@nwafu.edu.cn (J.W.); lwy1222@nwafu.edu.cn (W.L.); 2025056042@nwafu.edu.cn (S.L.); 13834511678@163.com (W.H.); 18617724471@163.com (S.L.); zijuntang@pku.edu.cn (Z.T.); 2 Xinjiang Research Institute of Agriculture in Arid Areas, Urumqi 830091, China; 3 College of Geomatics, Xi’an University of Science and Technology, Xi’an 710054, China

**Keywords:** soybean, hyperspectral, SPAD, vertical canopy heterogeneity, fractional-order differentiation, machine learning

## Abstract

Vertical heterogeneity in soybean canopies may lead to differences in the relationships between SPAD values at different canopy positions and canopy hyperspectral information, thereby affecting prediction performance. To characterize these spectral relationships and compare SPAD predictability, field experiments were conducted during the soybean flowering stage in Yangling, Shaanxi, China, in 2022 and 2023, with nine irrigation–nitrogen combinations and a rainfed control. A total of 120 paired observations of plant-level SPAD and canopy reflectance spectra were obtained from 20 fixed plots. Upper-layer SPAD, lower-layer SPAD, and their equal-weight arithmetic mean were used as estimation targets. Difference indices (DIs), soil-adjusted vegetation indices (SAVIs), and their combined feature set (ZU) were derived from fractional-order differentiated spectra and used with random forest (RF), support vector regression (SVR), and backpropagation neural network (BPNN) models to compare prediction performance among the three targets. The optimal differentiation orders and wavelength combinations varied among SPAD targets. Upper-layer SPAD showed its strongest association at an order of 0.4 (maximum |r| = 0.737), whereas lower-layer and mean SPAD showed their strongest associations at an order of 1.2, with maximum |r| values of 0.733 and 0.810, respectively. Across the spectral input–algorithm combinations, mean SPAD generally achieved higher prediction accuracy. Its best out-of-fold R^2^ was 0.636, with an RMSE of 2.450 SPAD units, compared with maximum R^2^ values of 0.461 and 0.510 for upper- and lower-layer SPAD, respectively. Further evaluation showed that mean-SPAD models maintained relatively favorable performance under repeated grouped validation and bidirectional cross-year testing. The SAVI-RF model achieved a repeated-validation R^2^ of 0.597 ± 0.064 and cross-year R^2^ values of 0.655 and 0.611 in the two directions. These findings indicate that SPAD predictability from canopy hyperspectral data varies with the sampling target, with the equal-weight mean of upper- and lower-layer SPAD showing relatively higher and more stable predictive performance within the current experiment. The study highlights the importance of vertical canopy sampling in hyperspectral SPAD estimation and provides evidence to inform sampling design and hyperspectral monitoring of soybean at flowering.

## 1. Introduction

Soybean (*Glycine max* L.) is an important food and oil crop [1], and monitoring its growth and nutritional status is essential for field management. Chlorophyll participates in photosynthesis, and its content provides useful information about plant nutritional status [2]. SPAD readings are widely used as a nondestructive indicator of relative leaf chlorophyll status [3]. However, these measurements are collected at individual leaf locations, which limits their efficiency for monitoring large areas [2]. Remote sensing therefore provides an important means of estimating chlorophyll-related crop traits rapidly and nondestructively over larger spatial scales [4].

Hyperspectral observations provide a basis for estimating chlorophyll-related plant parameters through their contiguous spectral bands and detailed spectral information. Previous studies have estimated soybean chlorophyll-related parameters using spectral indices, spectral transformations, and machine learning methods. For example, Shi et al. [5] compared optimized spectral indices and estimation models for soybean at different growth stages, while Gao et al. [6] investigated soybean leaf chlorophyll estimation by combining spectral resampling with machine learning. These studies provide methodological references for soybean spectral monitoring. However, canopy reflectance is influenced not only by leaf pigment content but also by canopy structure, leaf water content, background conditions, and illumination and viewing geometry [7]. Therefore, spectral estimation results should be interpreted in relation to the specific measurement target and observation conditions.

Crop canopies exhibit vertical heterogeneity. Leaves at different positions may differ in light environment, leaf age, and physiological status, contributing to variation in chlorophyll distribution. Bi et al. [8] used hyperspectral LiDAR to estimate chlorophyll concentrations at different vertical positions in maize, while Xu et al. [9] mapped three-dimensional plant chlorophyll distribution by combining hyperspectral LiDAR with a leaf–canopy radiative transfer model. These studies highlight the importance of considering spatial variation in chlorophyll when interpreting plant spectral observations. For soybean, differences between upper- and lower-layer leaves should likewise be considered, and measurements from a single canopy layer should not be directly equated with whole-plant chlorophyll status.

From the perspective of spectral observation, leaves at different canopy positions may contribute unequally to the observed signal because of differences in illumination, occlusion, leaf arrangement, and background conditions [7,9]. Consequently, using the same canopy spectra to predict upper-layer SPAD, lower-layer SPAD, or their arithmetic mean may yield different associations and predictive performance. Integrating measurements from multiple canopy layers may produce a more predictable target, while averaging may also reduce some random measurement variation when errors are not perfectly correlated. Whether mean SPAD is more predictable, and whether this advantage persists under different validation conditions, therefore requires evaluation using consistent data and modeling procedures. It cannot be inferred solely from the integrated nature of the canopy signal.

Spectral preprocessing and index construction are important steps in extracting information for prediction. Fractional-order differentiation modifies the representation of overall spectral trends and local variations by adjusting the differentiation order, providing flexible candidate transformations for spectral feature extraction. Previous studies have applied this approach to estimating leaf dry matter content [10] and chlorophyll-related parameters [11,12]. However, differentiation may amplify noise as well as highlight local spectral variations, and its predictive benefits should be evaluated for the specific input data, target variable, and modeling procedure. A higher differentiation order or a stronger correlation obtained from the full dataset should therefore not be assumed to yield better predictive performance. Differentiation orders, wavelength combinations, and model validation results should be considered together.

Spectral indices such as DI and SAVI combine information from different wavelengths and can serve as inputs to machine learning models for soybean chlorophyll estimation [5]. SAVI was originally developed for reflectance data to reduce soil background effects [13]. Applying these index formulations to differentiated spectra changes their numerical properties; their original physical interpretations therefore require reassessment rather than being assumed to remain unchanged. Whether combining multiple index types provides additional predictive information, whether normalization improves prediction relative to direct differencing, and how alternative spectral processing workflows perform should be evaluated using the same data and validation conditions. A literature-adapted baseline provides a reference for assessing the relative predictive performance and applicability of the proposed workflows.

The validation strategy also affects these evaluations. Screening across multiple differentiation orders and wavelength combinations involves a large candidate search space. If features are selected using the complete dataset before model evaluation, information from the validation samples may enter the modeling process, leading to optimistic performance estimates [14]. In addition, observations from plants within the same experimental plot and repeated observations across years may be dependent. Randomly assigning individual observations to training and validation sets without accounting for this structure may underestimate prediction error [15]. Feature selection and hyperparameter tuning should therefore be performed within the training data using a nested validation procedure [14,16], while data grouping should reflect the experimental structure and intended prediction task [15]. Repeated grouped validation and cross-year testing can further assess sensitivity to sample partitioning and year-to-year variation, providing additional evidence for interpreting model performance.

Based on these considerations, this study used paired plant-level spectral and SPAD observations collected during the flowering stage of soybean in 2022 and 2023. Upper-layer SPAD, lower-layer SPAD, and the equal-weight arithmetic mean of the two layers were used as the prediction targets. Fractional-order differentiation, spectral indices, and machine learning methods were combined to conduct the analysis. The specific objectives were to: (1) analyze the relationships between spectral indices and each SPAD target at different differentiation orders and identify the optimal orders and wavelength combinations under the current dataset; (2) compare the prediction performance of different SPAD targets and spectral inputs under consistent modeling and validation conditions; and (3) assess model stability through repeated grouped validation and bidirectional cross-year testing, with a literature-adapted baseline providing a supplementary performance reference. This study provides empirical evidence for integrating vertical sampling information, selecting SPAD estimation targets, and evaluating modeling methods in hyperspectral monitoring of flowering-stage soybean under similar experimental conditions.

## 2. Materials and Methods

### 2.1. Study Site

**Figure 1:**
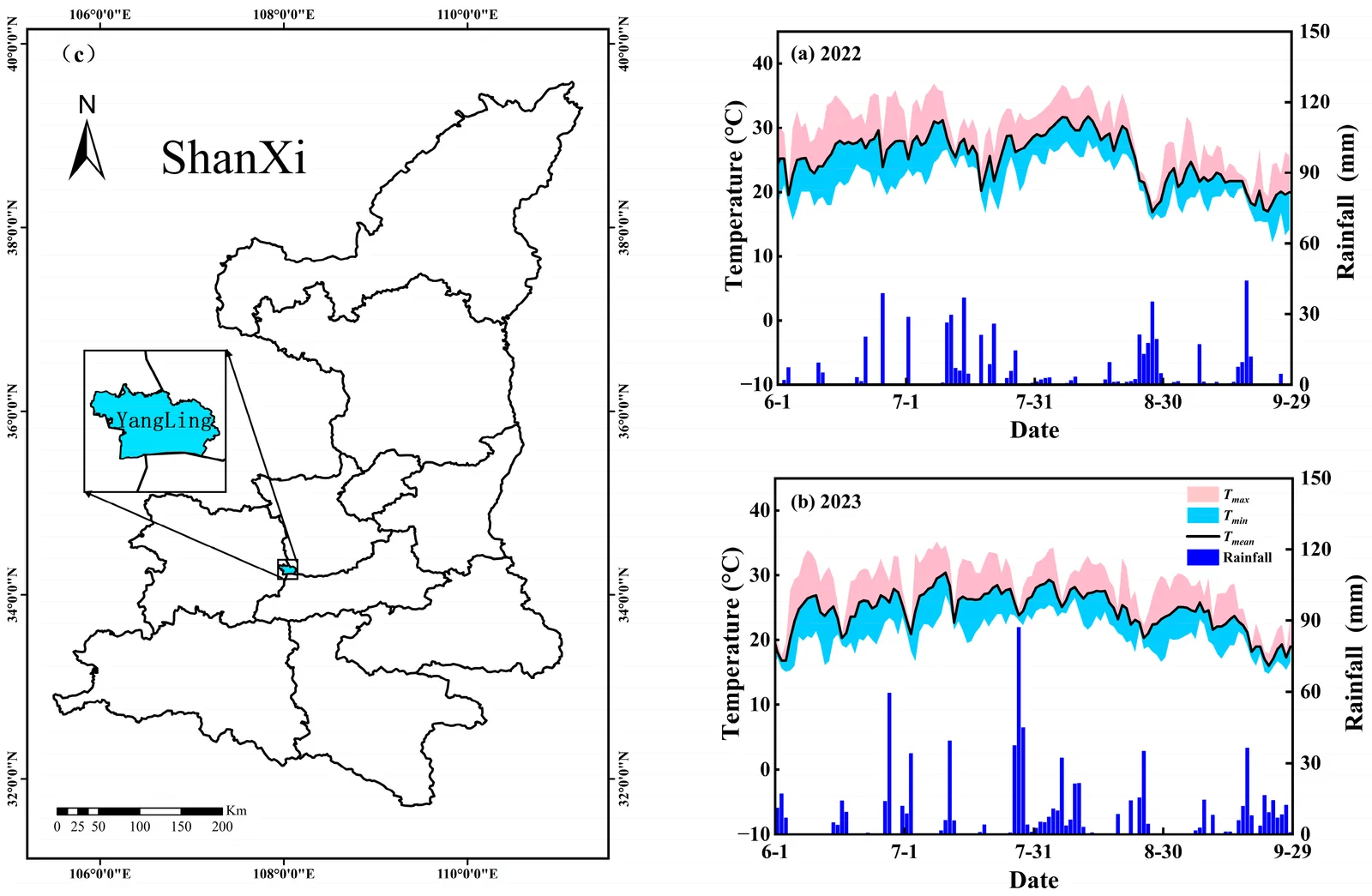
The location of the study area and the meteorological conditions, including daily maximum temperature (*T_max_*), minimum temperature (*T_min_*), mean temperature (*T_mean_*), and precipitation, during the soybean growing seasons from 2022 to 2023: (**a**) Meteorological conditions in 2022; (**b**) Meteorological conditions in 2023; (**c**) Location of the study area.

The field experiment was conducted at the Water-Saving Irrigation Experimental Station of the Key Laboratory of Agricultural Soil and Water Engineering in Arid and Semiarid Areas, Ministry of Education, Northwest A&F University, Yangling, Shaanxi Province, China (108°24′ E, 34°20′ N; altitude: 524.7 m). The study area is characterized by a warm temperate semi-humid monsoon climate, with concentrated precipitation occurring from July to September. The long-term average annual precipitation and air temperature are approximately 580 mm and 12.9 °C, respectively. The study site location and meteorological conditions during the growing seasons are shown in Figure 1.

The soybean cultivar ‘Shanning 17’ was used in this study. Field experiments were conducted during the 2022 and 2023 growing seasons, with sowing in June and harvesting in late September in both years. Three irrigation levels were established: W1 (50% *ET_c_*), W2 (75% *ET_c_*), and W3 (100% *ET_c_*), together with three nitrogen application rates: N1 (30 kg ha^−1^), N2 (60 kg ha^−1^), and N3 (90 kg ha^−1^). Nitrogen fertilizer was applied through a drip-fertigation system. Drip tapes were arranged between soybean rows, with a diameter of 1.6 cm, an emitter spacing of 30 cm, and a nominal discharge rate of 2 L h^−1^ under a working pressure of 0.1 MPa. For each plot, the fertilizer amount was calculated according to the target nitrogen rate and plot area. Fertigation followed a 1/4–1/2–1/4 procedure, consisting of pre-flushing with water, fertilizer application, and post-flushing with water. The same drip-tape specifications and fertigation procedure were used for all treatments to reduce differences in nitrogen delivery among plots. These treatments formed nine irrigation–nitrogen combinations, with an additional rainfed control (CK). Treatments were randomly assigned, with two replicates per treatment. Each plot covered 13.5 m^2^, giving a total of 20 experimental plots. No apparent pest or disease damage was observed during the soybean growing seasons.

Crop evapotranspiration (*ET_c_*) was calculated as the product of reference evapotranspiration (*ET_0_*) and the crop coefficient (*K_c_*), following the FAO-56 framework [17]:

*ET_c_* = *ET*_0_ × *K_c_* (1)

where *ET_0_* is the reference evapotranspiration, calculated using the FAO-56 Penman–Monteith equation [17]:

(2) $${\mathrm{ET}}_{0}=\frac{0.408\Delta (R_{n}-G)+\gamma \frac{900}{T_{\mathrm{mean}}+273}u_{2}(e_{s}-e_{a})}{\Delta +\gamma (1+0.34u_{2})}$$

where *R_n_* is the net radiation at the crop surface, *G* is the soil heat flux, *T_mean_* is the mean daily air temperature, *u_2_* is the wind speed at a height of 2 m, *e_s_* − *e_a_* is the vapor pressure deficit, Δ is the slope of the saturation vapor pressure curve, and *γ* is the psychrometric constant. Stage-specific crop coefficients (*K_c_*) were used for the corresponding growth stages.

### 2.2. SPAD Measurements

SSPAD measurements were conducted during the soybean flowering stage on 26 July 2022 and 28 July 2023, with one sampling day in each year. Three representative plants were selected from each of the 20 experimental plots, yielding 60 plant-level observations per year and 120 observations in total. Upper- and lower-layer leaves were selected according to their relative vertical positions on each plant. Three representative trifoliate leaves were selected per layer, and only the central leaflet of each trifoliate leaf was measured. The two layers were defined operationally rather than by fixed leaf rank or quantitative plant-height thresholds. This procedure was used consistently for all plant-level observations. Measurements were taken using a portable chlorophyll meter (SPAD-502, Konica Minolta, Tokyo, Japan). Three readings were taken at different points on each central leaflet and averaged arithmetically. The resulting means for the three central leaflets within each layer were then averaged to obtain the upper- or lower-layer SPAD value for the corresponding plant.

Mean SPAD was defined as the equal-weight arithmetic mean of the upper- and lower-layer SPAD values from the same plant. This metric was calculated from measurements of representative leaves in the two layers and was not equivalent to the mean SPAD of all leaves on the plant. Each plant-level SPAD observation was paired with the spectral record acquired for the same plant during the same measurement session. Observations from the three plants within each plot were retained separately rather than averaged into a single plot-level observation.

Each physical plot contributed six plant-level observations over the two years. To account for potential within-plot dependence among the 120 observations, model validation was grouped by physical plot [15], as described in Section 2.6. Table 1 summarizes the statistical characteristics of upper-layer, lower-layer, and mean SPAD.

### 2.3. Canopy Hyperspectral Reflectance Measurements

Spectral data were acquired using a FieldSpec^®^ 4 portable spectroradiometer (Analytical Spectral Devices Inc., Boulder, CO, USA). The spectra used in this study covered 350–1830 nm at a wavelength interval of 1 nm, comprising 1481 bands. The 1 nm interval represents the wavelength sampling grid of the input data and does not correspond to the instrument’s actual spectral resolution.

Canopy hyperspectral reflectance and SPAD measurements were collected on the same sampling day, on 26 July 2022 and 28 July 2023, respectively. Spectral measurements were conducted between 10:00 and 14:00 local time on each sampling day.

During measurements, the fiber-optic probe was positioned vertically downward approximately 1 m above the canopy. With a 25° field of view, the nominal observation footprint at canopy-top level was approximately 0.44 m in diameter. Three representative plants were selected in each plot, and a spectral sampling point was centered on each plant. Nine consecutive spectra were collected at each sampling point and averaged arithmetically to obtain a single spectral record, which was paired with the upper-layer, lower-layer, and mean SPAD values of the corresponding plant from the same measurement session. The canopy reflectance spectra were collected with the field of view centered on each target plant; the observed area could also include leaves from neighboring plants. A total of 120 paired spectral and SPAD observations were obtained over the two years, and the plant-level records were used as inputs for subsequent spectral index calculations and modeling analyses.

### 2.4. Hyperspectral Data Processing

Spectral index calculations and model validation were performed using MATLAB R2024a (The MathWorks, Inc., Natick, MA, USA). Figure preparation and graphical analyses were conducted using Origin 2021 (OriginLab Corporation, Northampton, MA, USA). The representative spectral curves in Supplementary Figure S2 were plotted using Python 3.13.7 and Matplotlib 3.10.8.

Previously smoothed spectra were used as inputs for fractional-order differentiation using the discrete Grünwald–Letnikov (G–L) method [18]. Differentiation orders ranged from 0.2 to 2.0 in increments of 0.2, yielding 10 orders. The smoothed spectra served as inputs to differentiation and were not included among the main differentiation-order results discussed in this study. For an equally spaced spectral sequence, the discrete approximation of G–L fractional-order differentiation is expressed as:

(3) $$D^{\alpha }R(\lambda_{k})\approx \frac{1}{h^{\alpha }}\sum_{j=0}^{k}{w_{j}}^{\left(\alpha \right)}R(\lambda_{k-j})$$

The weights were calculated recursively as:

(4) $${w_{0}}^{\left(\alpha \right)}=1,{w_{j}}^{\left(\alpha \right)}=(1-\frac{\alpha +1}{j}){w_{j-1}}^{\left(\alpha \right)},j=1,2,\ldots ,k$$

where *R*(*λ_k_*) is the smoothed spectral value at wavelength *λ_k_*; *α* is the differentiation order; *h* is the wavelength sampling interval, set to 1 nm in this study; *w_j_*^(*α*)^ is the weight of the *j*th term; and *k* is the wavelength sequence index, starting from 0. Calculations proceeded in ascending wavelength order, using the spectral values at the current wavelength and all available preceding wavelengths.

Fractional-order differentiation can alter the representation of overall spectral trends and local variations, but it may also amplify noise. Accordingly, differentiated spectra were used to construct candidate modeling configurations, and increases in correlation coefficients were not directly interpreted as improvements in the signal-to-noise ratio.

Representative input reflectance spectra and their 0.4- and 1.2-order differentiated spectra are shown in Supplementary Figure S2. Within each year, measured mean SPAD was divided into three groups using its one-third and two-thirds quantiles. The observation closest to the median of each group was selected, with ties resolved by sample ID order, yielding six observations. Sample IDs, SPAD values, and grouping boundaries are provided in Supplementary Table S14. Selection did not use correlations, prediction errors, or model performance.

### 2.5. Spectral Index Construction

The difference index (DI), soil-adjusted vegetation index (SAVI), and their combined feature set (ZU) were used as the primary model inputs. The normalized difference index (NDI) was additionally included as a supplementary comparator to DI to assess whether normalizing the difference between two spectral bands by their sum improved SPAD prediction. NDI was neither included in the construction of ZU nor considered in the selection of the best-performing DI, SAVI, and ZU models. The index formulas are provided in Table 2.

DI and SAVI were selected as candidate spectral indices based on their application to soybean chlorophyll estimation [5]. DI expresses the difference between spectral values at two wavelengths. SAVI was originally developed for reflectance data to reduce soil background effects [13]. In this study, these formulations were applied to differentiated spectra to construct candidate predictive features; their original physical interpretations were not assumed to remain unchanged. NDI was included as a supplementary comparator using the difference between two spectral values divided by their sum, without restricting the wavelength pair to the red and near-infrared bands conventionally used in NDVI.

Pearson’s correlation coefficient (*r*) was used to assess the linear associations between candidate indices and upper-layer, lower-layer, and mean SPAD. Features were selected according to the absolute correlation coefficient. Correlation matrices calculated from the full dataset were used only for exploratory description, retaining the signs of the correlation coefficients. Significance thresholds without adjustment for multiple comparisons were not used to identify optimal combinations. During model validation, feature selection was performed independently within the corresponding training folds, rather than directly using the optimal combinations identified from the full-data correlation matrices, to avoid selection-induced bias [14].

For DI and SAVI, the wavelength pair with the highest absolute correlation coefficient was first selected at each of the 10 differentiation orders ranging from 0.2 to 2.0 within each training dataset. The five features with the strongest correlations were then selected from the resulting 10 candidates as model inputs. For ZU, the DI and SAVI candidate features were pooled, and the five features with the strongest correlations were selected. Each feature was defined jointly by its index type, differentiation order, and wavelength pair. NDI followed the same feature-selection procedure for differentiated spectra and used the same inner and outer data partitions and candidate model hyperparameters as DI.

Because differentiated spectra can contain negative values, the denominator of NDI may approach zero. To control numerical anomalies, wavelength pairs were excluded from feature selection if any training observation satisfied *|R_i_ + R_j_|* ≤ 10^−10^. If a selected wavelength pair produced a denominator satisfying this threshold or a non-finite NDI value in the held-out data, the affected value was replaced with the median value of that feature calculated from the corresponding training data, and the number of replacements was recorded. This procedure served only as a numerical safeguard and did not guarantee the elimination of instability caused by small denominators. Because wavelength selection was performed separately for DI and NDI, their comparison reflects differences between their respective modeling workflows; performance differences were not attributed solely to normalization.

DI and NDI used identical sample partitions, numbers of input features, modeling algorithms, and candidate hyperparameters, with features selected independently within the corresponding training folds. This comparison evaluated the separately optimized workflows for the two indices rather than constituting an ablation analysis of normalization using fixed wavelength combinations.

### 2.6. SPAD Estimation Model Development and Validation

Random forest (RF) [19], support vector regression (SVR) [20], and backpropagation neural network (BPNN) [21] models were developed using upper-layer, lower-layer, and mean SPAD as target variables and the DI, SAVI, and ZU feature sets as inputs. Each configuration used five features, with feature selection performed independently within the corresponding training data.

(1) Model Settings

RF used 100 regression trees, with candidate minimum leaf sizes of 3 and 5. One input feature was randomly selected for consideration at each node split. SVR used a Gaussian kernel, with the kernel scale parameter (KernelScale in MATLAB R2024a) fixed at 5, candidate penalty parameter C values of 1 and 20, and the ε-insensitive loss parameter set to 0.1. BPNN had a single hidden layer with either 3 or 5 neurons. The hidden and output layers used hyperbolic tangent sigmoid and linear activation functions, respectively. The network was trained using the Levenberg–Marquardt algorithm [22], with a regularization parameter of 0.1 and a maximum of 200 training epochs.

Input features were standardized using means and standard deviations calculated exclusively from the current training data, and the same transformation was applied to the corresponding held-out data. The BPNN target variable was also scaled using the mean and standard deviation of the current training data, and predictions were subsequently transformed back to the original SPAD scale. The default internal random data partitioning of BPNN was disabled to avoid conflicts with the predefined grouped validation procedure.

(2) Plot-Grouped Nested Cross-Validation

A plot-grouped nested cross-validation framework was used to account for within-plot dependence and separate hyperparameter selection from performance evaluation [15,16]. The 20 physical plots were divided into five outer folds, with all plant observations from both years within each plot assigned to the same fold. In each iteration, 96 observations from 16 plots were used for training, and the remaining 24 observations from four plots were used for validation. This ensured that observations from the same plot did not appear in both the training and validation data within a given iteration.

Within each outer training set, three-fold cross-validation grouped by plot was performed. Feature selection, standardization, and model fitting were conducted independently within each inner training fold, followed by prediction for the corresponding inner held-out data. Model hyperparameters were selected based on the RMSE calculated from the pooled predictions across the three inner validation folds. After hyperparameter selection, features were selected again, standardization parameters were estimated, and the model was fitted using the entire outer training set. The fitted model was then used to predict the outer validation data. Outer validation data were not used for feature selection, estimation of standardization parameters, or hyperparameter selection [14,16].

After all five outer folds were completed, the validation predictions were combined to obtain 120 out-of-fold (OOF) predictions. Each observation received exactly one validation prediction from a model trained without that observation. The primary validation metrics were calculated from these 120 OOF predictions, whereas training metrics were reported as the arithmetic means of the metrics from the five outer training folds. Definitions of the evaluation metrics are provided in Section 2.7.

(3) Repeated Grouped Validation and Cross-Year Testing

To assess sensitivity to sample partitioning, four additional grouped partitions were generated alongside the original fixed five-fold plot partition, resulting in five repetitions of grouped nested cross-validation. The original partition used the random seed 20260920, and the additional partitions used seeds 20260921–20260924, respectively. The complete sample assignments for all partitions were retained. Model random seeds were set to 20260920 plus 100 times the outer fold index, with the inner fold index additionally included during inner-fold model fitting. Evaluation metrics were calculated separately for each repetition and summarized as means and standard deviations. Because all repetitions reused the same observations, the standard deviations describe performance variability associated with partitioning and should not be interpreted as confidence intervals based on independent experiments.

Bidirectional cross-year testing was also conducted by training on the 60 observations from 2022 and testing on the 60 observations from 2023, followed by reversing the training and testing years. Feature selection, standardization, and inner grouped hyperparameter tuning were restricted to the training-year data. Because observations from both years originated from the same site and physical plots, this analysis assessed temporal transferability within the existing experimental setting rather than external validation using independent sites or plots.

(4) Model Comparison and Supplementary Comparisons

The predictive performance of RF, SVR, and BPNN was compared for each SPAD target and input feature set. For each target, Figure 5 presents the algorithm achieving the highest OOF R^2^ for DI, SAVI, and ZU under the original fixed plot-grouped five-fold partition, resulting in nine panels, each containing 120 OOF predictions. This selection illustrates the comparative performance of the candidate models and does not represent optimal model performance confirmed by independent testing. Model robustness was evaluated using repeated grouped validation and cross-year testing.

NDI was included only as a supplementary comparator to DI, using identical inner and outer sample partitions, numbers of features, candidate model hyperparameters, and evaluation metrics. NDI was neither included in ZU nor considered in the selection of the primary models.

A literature-adapted baseline was constructed based on Shi et al. [5]. It used the same observations, original fixed plot-grouped nested cross-validation partitions, and candidate hyperparameters for the corresponding algorithms as the primary workflows, with feature selection and preprocessing parameter estimation restricted to the training data. Baseline-specific spectral preprocessing and index definitions are provided in Supplementary Table S1 and the accompanying methods. The baseline was also evaluated using bidirectional cross-year testing, but not the four additional grouped validation repetitions. This comparison evaluated complete modeling workflows and did not isolate the effect of fractional-order differentiation or constitute a complete reproduction of the original study. The baseline included additional Savitzky–Golay filtering [23] followed by first-order backward differencing; implementation details are provided in the Supplementary Materials.

Residual diagnostics were conducted using OOF predictions from the original fixed plot-grouped five-fold validation, with residuals defined as predicted minus measured values. Observations were divided into quartile-based groups according to measured SPAD for each target, and the mean bias and RMSE were calculated within each group to describe how prediction errors varied across the measured SPAD range. This analysis was not used to exclude observations or reselect models.

### 2.7. Model Evaluation and Correlation Analysis

(1) Model Evaluation Metrics

Model performance was evaluated using the coefficient of determination (*R*^2^), root mean square error (*RMSE*), normalized root mean square error (*NRMSE*), and mean relative error (*MRE*), calculated as follows:

(5) $$R^{2}=1-\frac{\sum_{i=1}^{n}{\left(y_{i}-{\hat{y}}_{i}\right)}^{2}}{\sum_{i=1}^{n}{\left(y_{i}-\overset{-}{y}\right)}^{2}}$$

(6) $$\mathrm{RMSE}=\sqrt{\frac{\sum_{i=1}^{n}{\left(y_{i}-{\hat{y}}_{i}\right)}^{2}}{n}}$$

(7) $$\mathrm{MRE}=\frac{1}{n}\sum_{i=1}^{n}\left|\frac{y_{i}-{\hat{y}}_{i}}{y_{i}}\right|\times 100\%$$

(8) $$\mathrm{NRMSE}=\frac{\mathrm{RMSE}}{\overset{-}{y}}\times 100\%$$

where *y_i_* is the measured SPAD value, *ŷ_i_* is the corresponding predicted value, *ȳ* is the mean measured SPAD value of the current evaluation set, and *n* is the number of observations in that set. *R*^2^ may also take negative values.

The primary validation metrics were calculated from the 120 unique out-of-fold predictions obtained by pooling the predictions from the five outer validation folds; R^2^ was not calculated as the arithmetic mean of the R^2^ values from the five validation folds. Training metrics were reported as the arithmetic means of the corresponding metrics from the five outer training folds. For repeated grouped validation, the metrics were calculated separately for each partition and then reported as means and standard deviations. For bidirectional cross-year validation, the metrics were reported separately for the two training–testing directions.

(2) Exploratory Correlation Analysis

Pearson’s correlation coefficient r was used to describe the linear associations between the spectral indices and upper-layer, lower-layer, and mean SPAD. The correlation coefficient was calculated as follows:

(9) $$r=\frac{\sum_{i=1}^{n}\left(x_{i}-\overset{-}{x})(y_{i}-\overset{-}{y}\right)}{\sqrt{\sum_{i=1}^{n}{\left(x_{i}-\overset{-}{x}\right)}^{2}\sum_{i=1}^{n}{\left(y_{i}-\overset{-}{y}\right)}^{2}}}$$

where *x_i_* is the spectral index value of the *i*th observation, and $\overset{-}{x}$ is its mean; *y_i_* and *ȳ* are the measured SPAD value and its mean, respectively. Feature selection and comparison of association strength were based on |r|, whereas the correlation matrices retained the signs of *r*. Pearson’s correlation coefficient is distinct from the predictive *R*^2^ defined above.

Because the analysis involved multiple differentiation orders and numerous wavelength combinations, and the sampling design introduced potential within-plot dependence [15], the full-data correlation matrices and optimal correlation-based combinations were treated as exploratory results. An unadjusted threshold of *p* < 0.01 was not used to establish the statistical significance of the selected combinations. During model validation, correlation-based feature screening was performed exclusively within the corresponding training folds to prevent information from the outer validation data from entering feature selection [14].

## 3. Results

### 3.1. Correlations Between Spectral Indices and SPAD and Optimal Wavelength Combinations

The correlations of DI and SAVI with upper-layer, lower-layer, and mean SPAD were analyzed at different differentiation orders using 120 paired spectral and SPAD observations. Differentiation orders ranged from 0.2 to 2.0 in increments of 0.2. Association strength was evaluated using the absolute Pearson correlation coefficient (|r|), and the wavelength pair yielding the highest |r| at each order was identified as the optimal correlation-based combination for that order.

**Figure 2:**
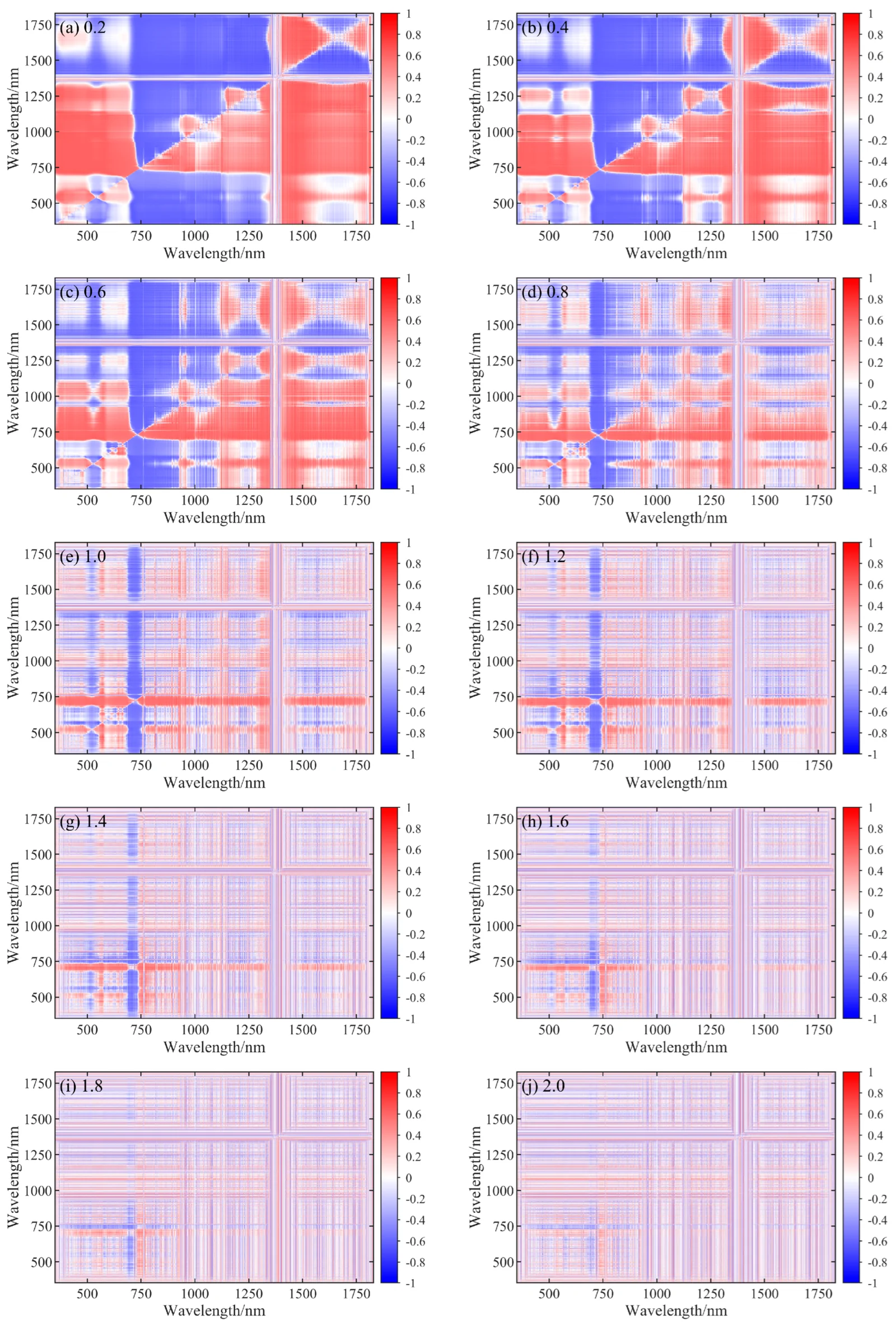
Correlation matrices between fractional-order differentiated spectral indices and upper-layer SPAD values (orders 0.2–2.0, in increments of 0.2; panels (**a**–**j**)). DI is shown above the diagonal and SAVI below the diagonal. The horizontal and vertical axes represent wavelengths j and i, respectively. Color shading indicates the signed Pearson correlation coefficient (*r*), whereas optimal wavelength combinations were selected according to |r|.

The correlation matrices and optimal wavelength combinations at each order for upper-layer SPAD are presented in Figure 2 and Table 3, respectively. Both DI and SAVI showed their strongest associations at an order of 0.4. Specifically, the optimal wavelength pair for DI-S was 1525 and 1578 nm, with |r| = 0.7318, whereas that for SAVI-S was 1525 and 1577 nm, with |r| = 0.7365. The optimal wavelengths for both indices were located in the shortwave infrared region, and their association strengths were similar. At an order of 1.6, the |r| values for DI-S and SAVI-S were 0.7097 and 0.7095, respectively, both lower than the corresponding values at an order of 0.4.

**Figure 3:**
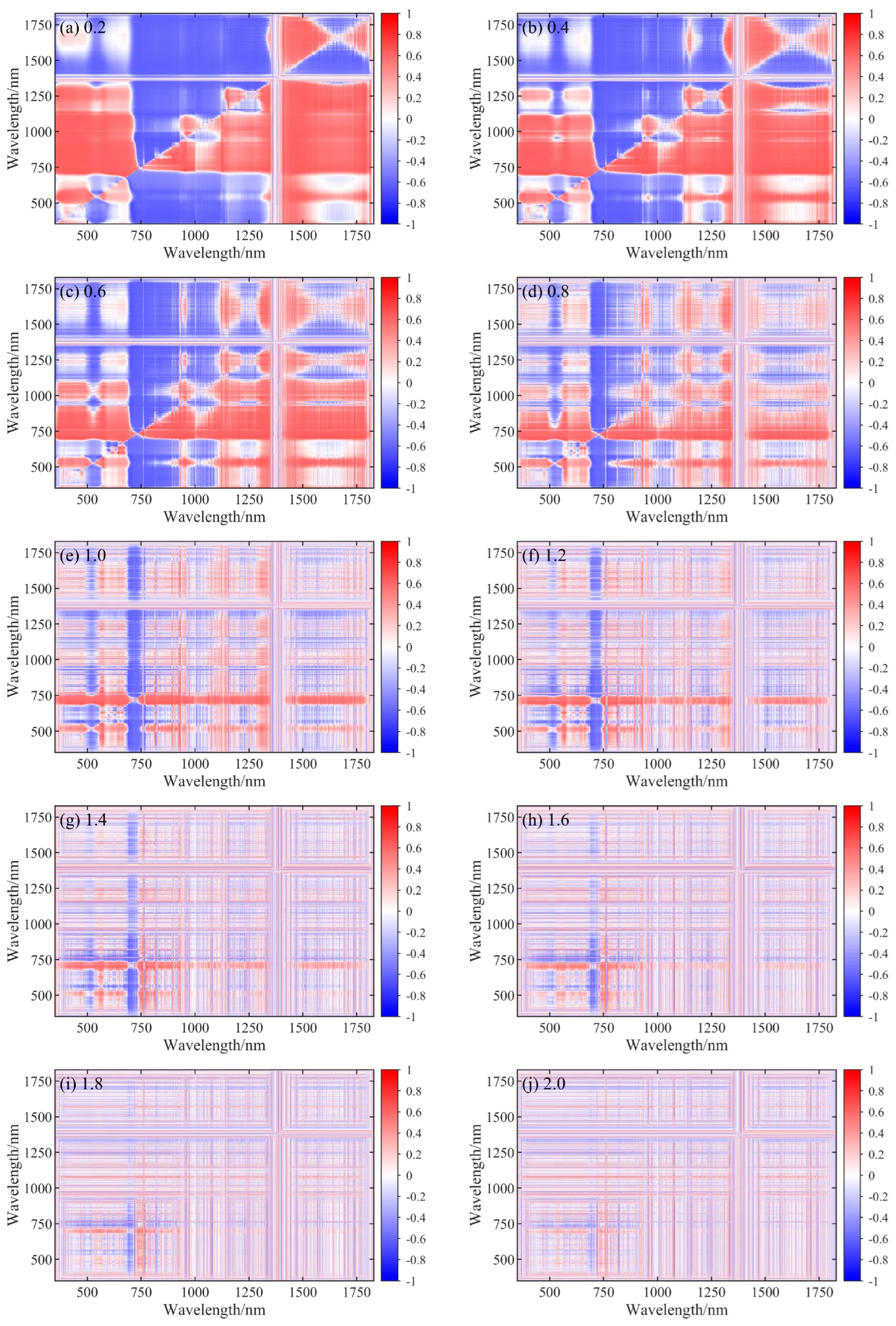
Correlation matrices between fractional-order differentiated spectral indices and lower-layer SPAD values (orders 0.2–2.0, in increments of 0.2; panels (**a**–**j**)). DI is shown above the diagonal and SAVI below the diagonal. The horizontal and vertical axes represent wavelengths j and i, respectively. Color shading indicates the signed Pearson correlation coefficient (*r*), whereas optimal wavelength combinations were selected according to |r|.

The correlation matrices and optimal wavelength combinations at each order for lower-layer SPAD are presented in Figure 3 and Table 4, respectively. Both DI and SAVI exhibited their strongest associations at an order of 1.2 using the wavelength pair of 691 and 1578 nm, with |r| values of 0.7329 and 0.7328, respectively. The maximum |r| for SAVI at an order of 0.6 was 0.7326, close to that at an order of 1.2, indicating that strong associations were not restricted to a single differentiation order. These comparisons describe numerical differences and do not establish statistically significant differences between orders.

**Figure 4:**
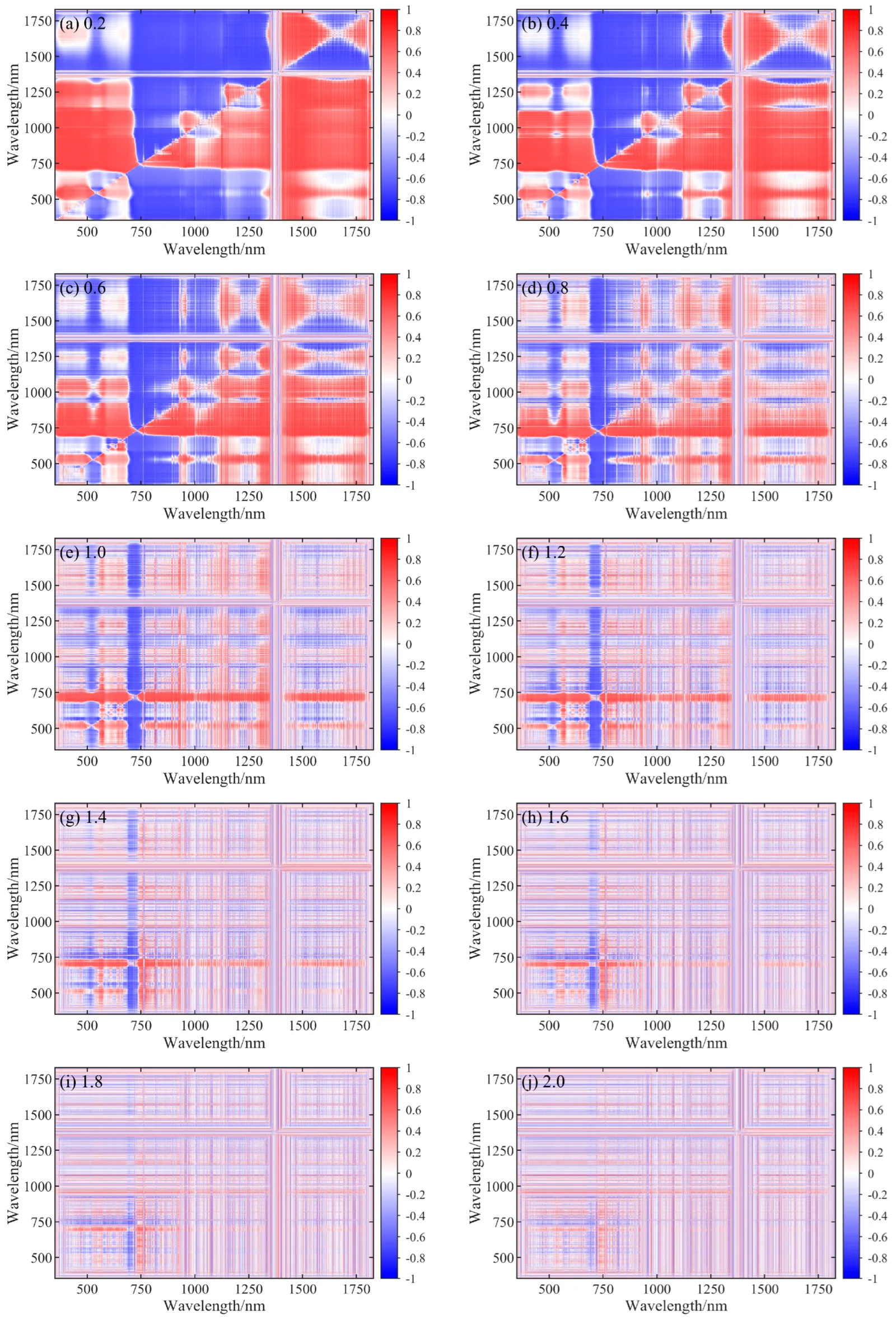
Correlation matrices between fractional-order differentiated spectral indices and the arithmetic mean of upper- and lower-layer SPAD values (orders 0.2–2.0, in increments of 0.2; panels (**a**–**j**)). DI is shown above the diagonal and SAVI below the diagonal. The horizontal and vertical axes represent wavelengths j and i, respectively. Color shading indicates the signed Pearson correlation coefficient (*r*), whereas optimal wavelength combinations were selected according to |r|.

The correlation matrices and optimal wavelength combinations at each order for mean SPAD are presented in Figure 4 and Table 5, respectively. Both DI and SAVI exhibited their strongest associations at an order of 1.2 using the wavelength pair of 691 and 1578 nm, with |r| values of 0.8100 and 0.8101, respectively. These maximum |r| values exceeded those for upper- and lower-layer SPAD, indicating that mean SPAD had stronger linear associations with the selected spectral indices within the current dataset and candidate search space. DI and SAVI showed similar maximum association strengths for this target.

Overall, the optimal differentiation orders and wavelength combinations varied among SPAD targets, and association strength did not increase consistently with differentiation order. The strongest associations occurred at an order of 0.4 for upper-layer SPAD and at an order of 1.2 for lower-layer and mean SPAD. These results describe the associations between spectral indices and SPAD across the full dataset. Because the optimal combinations were selected from a large number of candidates, their association strengths do not directly indicate model generalization performance, nor do they alone establish that mean SPAD is more physiologically representative.

### 3.2. Development and Comparison of SPAD Estimation Models Across Vertical Canopy Targets

BPNN, SVR, and RF models were developed to estimate upper-layer, lower-layer, and mean SPAD using DI, SAVI, and their combined feature set ZU as inputs. Model performance was evaluated using nested cross-validation grouped by fixed plot. The training metrics in Table 6, Table 7 and Table 8 represent the means of the metrics from the five outer training folds, whereas validation metrics were calculated from the pooled out-of-fold predictions for all 120 observations. Each validation observation was excluded from feature selection, hyperparameter tuning, and training of its corresponding model. The following comparisons are based on out-of-fold validation results, with NDI included only as a supplementary comparator to DI.

The estimation results for upper-layer SPAD are presented in Table 6. For DI, SAVI, and ZU, RF achieved higher validation R^2^ values than the corresponding BPNN and SVR models, with values of 0.457, 0.461, and 0.459, respectively. Among these models, SAVI-S-RF achieved the highest validation R^2^, with an RMSE of 3.105, an NRMSE of 6.373%, and an MRE of 4.838%. The RF models using the three input feature sets showed similar validation performance, with relatively small numerical differences under the current dataset and validation scheme.

The estimation results for lower-layer SPAD are presented in Table 7. Unlike upper-layer SPAD, BPNN achieved the highest validation R^2^ for each of the DI, SAVI, and ZU input feature sets, with values of 0.483, 0.510, and 0.504, respectively. Among these models, SAVI-X-BPNN achieved the highest validation R^2^, with an RMSE of 3.509, an NRMSE of 7.337%, and an MRE of 5.209%. ZU-X-BPNN performed similarly but did not outperform SAVI-X-BPNN. These results indicate that model performance differed between lower- and upper-layer SPAD, with the best-performing algorithm varying according to the estimation target.

The estimation results for mean SPAD are presented in Table 8. Across the DI, SAVI, and ZU input feature sets, validation R^2^ values ranged from 0.569 to 0.636 and were consistently higher than those of the upper- and lower-layer SPAD models using the same input feature set and algorithm, while RMSE and NRMSE were consistently lower. Both DI-J-RF and SAVI-J-RF achieved a validation R^2^ of 0.636, an RMSE of 2.450, and an NRMSE of 5.076%, indicating similar predictive performance; their MRE values were 3.810% and 3.894%, respectively. With ZU as the input, BPNN achieved the highest validation R^2^ of 0.622, with an RMSE of 2.496 and an NRMSE of 5.171%. Overall, ZU did not demonstrate a consistent advantage over inputs derived from a single index type.

**Figure 5:**
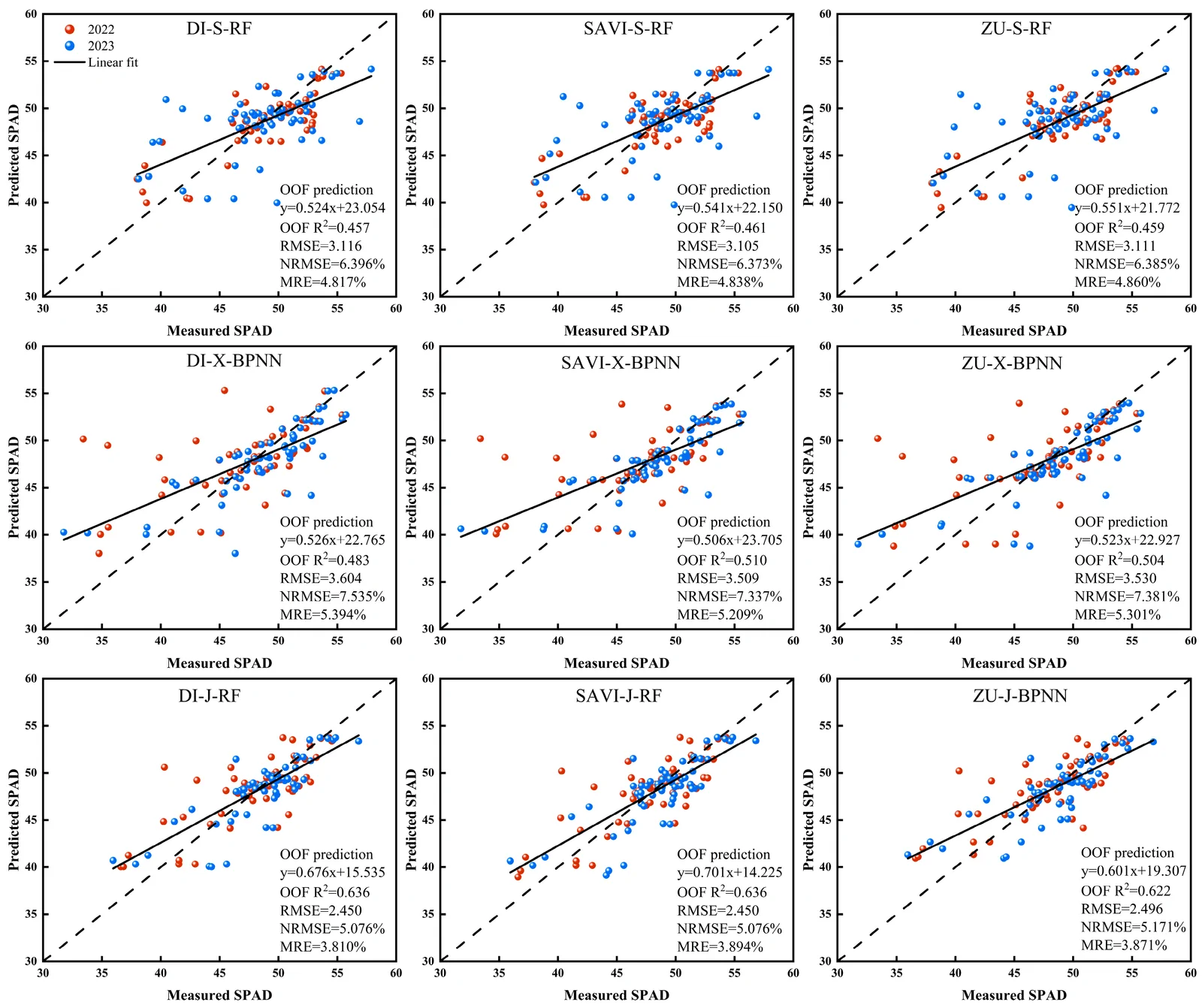
Measured and out-of-fold predicted values for the best-performing models for each SPAD target using different spectral index combinations. The two colors represent the two growing seasons, each comprising 60 out-of-fold predictions. The dashed line is the 1:1 reference line, and the solid line is fitted to all 120 pooled out-of-fold predictions and their corresponding measured SPAD values. Colors distinguish the 2022 (red) and 2023 (blue) growing seasons, with 60 observations per year, rather than training and validation sets. The fitted line is descriptive; model performance metrics were calculated directly from the measured and out-of-fold predicted values.

For each SPAD target and each of the DI, SAVI, and ZU input feature sets, the best-performing algorithm was selected based on validation R^2^. The relationships between measured and out-of-fold predicted values are shown in Figure 5. RF was selected for all three input feature sets for upper-layer SPAD, whereas BPNN was selected for all three for lower-layer SPAD. For mean SPAD, RF was selected for DI and SAVI, and BPNN for ZU. Each panel contains 120 out-of-fold predictions, with the two colors representing the 2022 and 2023 growing seasons, each comprising 60 observations. The dashed line denotes the 1:1 reference, whereas the solid line summarizes the relationship between all 120 pooled out-of-fold predictions and their corresponding measured values. The R^2^ values were calculated from the measured and out-of-fold predicted values using the definition in Section 2.7 and are consistent with the validation metrics in Table 6, Table 7 and Table 8. These nine configurations achieved the highest out-of-fold R^2^ among the candidate algorithms for their respective SPAD targets and input feature sets. Their reported performance remains a cross-validation assessment within the current dataset rather than an independent test estimate.

As a supplementary comparator to DI, the NDI estimation results are presented at the bottom of Table 6, Table 7 and Table 8. Under the current five-fold partition, BPNN achieved the highest validation R^2^ for upper-layer SPAD using NDI, at 0.380. For lower-layer and mean SPAD, RF achieved the highest validation R^2^ values of 0.478 and 0.611, respectively. Compared with DI models for the same target and algorithm, only NDI-X-RF achieved a slightly higher validation R^2^ than DI-X-RF; none of the other NDI models achieved higher validation R^2^ values than their DI counterparts. Therefore, the current results do not indicate a consistent predictive advantage of NDI over DI. This comparison is based on models with features selected separately for the two index types and does not isolate the effect of normalization using fixed wavelength combinations.

### 3.3. Model Validation Stability and Cross-Year Performance

To assess the sensitivity of model results to sample partitioning, four additional grouped partitions were introduced alongside the original fixed plot-grouped five-fold partition, resulting in five validation repetitions. Feature selection and hyperparameter tuning were performed within the training data for each repetition, and evaluation metrics were calculated from 120 out-of-fold predictions. Table 9 summarizes the repeated validation and cross-year testing results for the nine model configurations shown in Figure 5. The standard deviations from repeated validation describe performance variability caused by partitioning; they do not represent variability among independent experimental replicates or confidence intervals.

Across the five validation repetitions, mean R^2^ values ranged from 0.435 to 0.444 for the three upper-layer models, from 0.406 to 0.448 for the three lower-layer models, and from 0.550 to 0.597 for the three mean-SPAD models. SAVI-J-RF achieved an R^2^ of 0.597 ± 0.064 and an RMSE of 2.571 ± 0.198, whereas DI-J-RF achieved an R^2^ of 0.589 ± 0.074 and an RMSE of 2.598 ± 0.225 (mean ± standard deviation across five repetitions). The mean-SPAD models maintained relatively high average predictive performance across different partitions.

Cross-year testing produced different results in the two directions. Using SAVI as an example, the upper-layer RF model achieved an R^2^ of 0.189 when trained on 2022 data and tested on 2023 data, compared with 0.455 in the reverse direction. The corresponding R^2^ values for the lower-layer BPNN model were 0.552 and 0.279. The mean-SPAD SAVI-RF model achieved R^2^ values of 0.655 and 0.611 and RMSE values of 2.276 and 2.624 in the two directions, respectively, showing smaller differences in performance between the two testing directions than the corresponding upper- and lower-layer models. Because SPAD distributions differed between the two test years, differences in cross-year R^2^ should be interpreted alongside RMSE.

Residual diagnostics were further conducted using the out-of-fold predictions from the original fixed partition (Figure S1). Taking the mean-SPAD SAVI-RF model as an example, observations were grouped by quartiles of measured SPAD. The mean bias was 1.311 in the lowest SPAD interval and −1.358 in the highest interval (Table S10), indicating average overestimation at low values and underestimation at high values. Bias was defined as the predicted value minus the measured value. These patterns describe prediction errors conditional on measured SPAD and should not, on their own, be interpreted as evidence of physiological mechanisms or heteroscedasticity. All observations were retained during the diagnostic analysis, and none were excluded based on error magnitude.

The literature-adapted baseline showed that relative performance depended on the SPAD target and algorithm (Table S6). For lower-canopy SPAD, the DI, SAVI, and ZU workflows achieved numerically higher OOF R^2^ values than the corresponding baseline models using BPNN and RF; for example, SAVI-BPNN achieved an R^2^ of 0.510 compared with 0.466 for the baseline. Conversely, the baseline achieved the highest OOF R^2^ for upper-canopy SPAD using BPNN (0.492) and for mean SPAD using RF (0.670). Thus, the proposed workflows did not consistently outperform the adapted baseline. These comparisons were based on the original fixed partition and were descriptive; the baseline was not included in the additional grouped validation repetitions.

Overall, the mean-SPAD models maintained relatively favorable performance under repeated grouped validation and cross-year testing in both directions within the current dataset. However, both years used the same fixed plots at the same site. Cross-year testing therefore reflects temporal transferability within the existing experimental setting and does not replace independent external validation across sites and cultivars.

## 4. Discussion

### 4.1. Fractional-Order Differentiation and Extraction of SPAD-Related Spectral Features

Fractional-order differentiation modifies the representation of spectral features by adjusting the differentiation order, providing alternative transformations for extracting information related to plant biochemical parameters. Previous studies have applied this approach to estimating leaf dry matter content [10] and chlorophyll-related parameters at leaf and canopy scales [11,12]. Yang et al. [24] further compared three fractional-order derivative formulations for estimating winter wheat chlorophyll density and reported the best performance for a workflow combining a 0.3-order Riemann–Liouville derivative, wavelength selection, and extra-trees regression. Zhuang and Wang [25] also reported improved estimation of several leaf chlorophyll fluorescence parameters using indices derived from low-order fractional derivatives. These studies demonstrate potential applications of fractional-order spectral transformations, but their target variables, derivative formulations, and modeling procedures differ from those used here. Their optimal orders therefore cannot be transferred directly to soybean SPAD estimation.

In this study, both DI and SAVI exhibited their strongest correlations with upper-layer SPAD at an order of 0.4, whereas the strongest correlations with lower-layer and mean SPAD occurred at an order of 1.2. These findings indicate that different SPAD targets can favor different spectral transformations, even when derived from the same spectral dataset. However, the differences do not establish distinct physiological mechanisms. Furthermore, the maximum |r| values for SAVI and lower-layer SPAD at orders of 0.6 and 1.2 were 0.7326 and 0.7328, respectively. Their close agreement suggests that strong associations were not confined to a single order. The optimal orders reported here should thus be interpreted as numerical optima within the current dataset and search space rather than fixed recommendations for other experimental conditions.

Derivative analysis provides a means of emphasizing changes in spectral shape [26], but differentiation can also increase sensitivity to noise and the characteristics of the input spectra. In the present study, differentiation was applied to previously smoothed spectra sampled at 1 nm intervals. The identified orders therefore describe the behavior of this particular input and processing configuration. They do not establish that the same orders would remain optimal under different smoothing procedures or wavelength sampling intervals. Similarly, an increase in correlation after differentiation should not be interpreted as direct evidence of an improved signal-to-noise ratio.

A further consideration is the distinction between association screening and predictive evaluation. Searching across numerous orders and wavelength pairs makes the maximum observed correlations subject to selection effects. Feature selection must therefore be included within the validation procedure to avoid optimistic evaluation [14]. More generally, optimization over a finite dataset can introduce overfitting at the model-selection stage, even when performance is assessed using cross-validation [27]. Here, the full-data correlation matrices describe association patterns, whereas feature selection and hyperparameter tuning were repeated within the training folds for model evaluation [14,16]. The practical value of fractional-order differentiation should consequently be judged from out-of-fold performance, repeated validation, and comparison with the adapted baseline, rather than from the maximum full-data correlation alone.

### 4.2. Roles of Sensitive Wavelengths and Spectral Index Combinations

The optimal wavelengths identified in this study occurred in the red and shortwave infrared regions and cannot be explained solely by chlorophyll responses in the red-edge region. The strongest associations with upper-layer SPAD involved wavelengths within approximately 1525–1578 nm, whereas those for lower-layer and mean SPAD involved 691 and 1578 nm. The wavelength of 691 nm lies near the transition between red chlorophyll absorption and the red edge, making pigment-related variation a plausible contributor to its predictive value [28]. In contrast, reflectance in the shortwave infrared region is influenced by leaf water content as well as other leaf properties [29]. At the canopy scale, structural and background effects further contribute to reflectance variability [7,30]. These factors provide alternative explanations for the associations observed near 1525–1578 nm.

Soybean-specific evidence also supports considering water-related influences when interpreting spectral features. Crusiol et al. [31] investigated sensitive spectral bands for soybean water status monitoring and soil moisture prediction using leaf-level hyperspectral reflectance. Their study provides relevant context for considering water status but does not establish the mechanism underlying the 1578 nm feature in the present analysis. Under the current irrigation and nitrogen treatments, water status, leaf structure, and SPAD may covary, potentially giving shortwave infrared features indirect predictive value. Because these contributions were not separated experimentally, the selected wavelength combinations should be regarded as predictive features within the current dataset rather than established chlorophyll-specific sensitive bands.

Interpretation is further complicated by the spectral transformation itself. In the G–L implementation described in Section 2.4, differentiation at a given wavelength uses information from that wavelength and all available preceding wavelengths. Consequently, a selected wavelength identifies a position in the transformed spectrum rather than an isolated response of the corresponding original band. The combination of 691 and 1578 nm therefore cannot be interpreted simply as the independent contributions of a chlorophyll-sensitive band and a water-sensitive band. Establishing such a mechanism would require additional measurements or analyses that separate pigment, water, and structural effects.

DI and SAVI showed similar association strengths and predictive performance across several targets, whereas ZU did not demonstrate a consistent advantage. One possible explanation is that their candidate features contain overlapping information. Research on correlated predictors shows that dependence among input variables can complicate interpretation and affect transferability when predictor relationships change [32]. However, this does not establish feature redundancy as the cause of the present ZU results. Because ZU and the individual index configurations each contained five selected features, their comparison evaluates a change in candidate feature sources at a fixed input size. It does not test the effect of adding more predictors or demonstrate that combining spectral information is generally ineffective.

The physical interpretation of the index formulations also requires caution. SAVI was originally developed to reduce soil background effects in reflectance data [13]. Applying the same formulation to differentiated spectra changes the numerical properties of its inputs, so its original correction capability cannot be assumed to remain unchanged. Likewise, differentiated spectral values can be positive or negative, and the NDI denominator may approach zero, potentially amplifying numerical fluctuations. The numerical safeguards used here limit undefined or extreme calculations but do not establish improved resistance to illumination or background effects. Because DI and NDI underwent separate feature selection, their comparison reflects the performance of separately optimized workflows rather than the isolated effect of normalization at fixed wavelengths.

### 4.3. Vertical Canopy Differences and the Predictive Performance of Mean SPAD

Leaves at different canopy positions experience different light environments and may differ in age, structure, and physiological status. Direct evidence from soybean indicates that vertical differences in leaf properties depend on canopy development. Shiraiwa and Sinclair [33] found that the distribution of leaf nitrogen per unit area differed between soybean canopies containing expanding leaves and mature canopies with fully expanded leaves. Although leaf nitrogen is not equivalent to SPAD, these findings support considering canopy position and developmental status when interpreting soybean leaf measurements. Experiments across multiple species have also shown that growth irradiance affects specific leaf area and nitrogen partitioning [34], while modeling research has highlighted the need to distinguish leaf-age effects from light acclimation [35]. These studies provide physiological context for vertical heterogeneity but do not establish the mechanisms responsible for the SPAD patterns observed here.

Hyperspectral studies of vertical and three-dimensional chlorophyll distributions further demonstrate the relevance of spatial heterogeneity to spectral estimation [8,9]. However, its implications for model selection are less straightforward. In this study, RF achieved the highest validation R^2^ for upper-layer SPAD across the three primary input feature sets, whereas BPNN performed best for lower-layer SPAD. These differences may reflect interactions among target distributions, spectral features, sample size, and the hyperparameter search space. They should not be interpreted as evidence that a particular algorithm is universally suitable for a specific canopy position.

Among the primary DI, SAVI, and ZU models, mean SPAD consistently achieved higher validation R^2^ and lower RMSE and NRMSE than the individual layers when the input feature set and algorithm were held constant (Table 6, Table 7 and Table 8). Repeated validation and cross-year testing provided additional support for this pattern. For example, SAVI-J-RF achieved an R^2^ of 0.597 ± 0.064 across five validation repetitions and cross-year test R^2^ values of 0.655 and 0.611 in the two directions (Table 9). These results indicate that its favorable performance was not restricted to the original partition, but they do not directly establish greater physiological representativeness.

Two explanations may contribute to the predictive performance of mean SPAD. First, local canopy spectra integrate signals from multiple leaves and are influenced by canopy structure [7,30]. Combining upper- and lower-layer measurements may therefore provide a target that corresponds more closely to the integrated spectral signal. Second, averaging may reduce a component of random measurement error, depending on the error variances and covariance between layers. These explanations remain hypotheses: the present study did not quantify the contributions of individual canopy layers to the observed spectra or independently estimate measurement-error variance. Moreover, a reduction in target variance alone does not guarantee higher R^2^, which depends on prediction error relative to the total variation in the target.

The interpretation of mean SPAD also requires attention to the measurement scale. SPAD readings are optical indicators rather than direct measurements of chlorophyll mass [3]. Monje and Bugbee [36] identified leaf light scattering and nonuniform chlorophyll distribution as limitations of nondestructive chlorophyll meters. Parry et al. [37] further showed that the relationship between optical readings and absolute chlorophyll concentration can be nonlinear and vary among species. Consequently, an arithmetic mean of SPAD readings is not automatically equivalent to an arithmetic mean of absolute chlorophyll concentration. In this study, mean SPAD was the equal-weight mean of two sampled layers, without weighting by leaf number or leaf area. It should therefore be regarded as a defined sampling-based target with favorable predictability under the tested conditions, rather than a validated measure of whole-plant chlorophyll status or total canopy chlorophyll content.

### 4.4. Validation Scope and Study Limitations

The adapted baseline comparison showed no consistent predictive advantage of the proposed spectral workflows across SPAD targets and algorithms (Table S6). Because the workflows differed in preprocessing, wavelength coverage, and candidate indices, these results cannot isolate the contribution of fractional-order differentiation. The main contribution of this study is therefore the comparison of vertically defined SPAD targets under a common plot-grouped nested validation framework, with feature selection and tuning restricted to the training data, rather than evidence of universal superiority over existing approaches.

The experimental hierarchy is important when interpreting these results. The 120 plant-level observations originated from 20 fixed plots over two growing seasons and therefore do not constitute 120 independent experimental replicates. The distinction between experimental replication and subsampling is fundamental to avoiding pseudoreplication [38]. Here, observations from the same physical plot were kept together during grouped validation. This approach addresses potential within-plot dependence [15] and is consistent with the principle that validation design should reflect the intended prediction task [39]. Feature selection and hyperparameter tuning were additionally restricted to the training data to reduce information leakage [14,16]. These procedures strengthen the evaluation but do not increase the number of independent plots or establish generalization beyond the sampled experimental conditions.

Repeated grouped validation assessed sensitivity to the assignment of plots to folds. The observed variability is relevant because predictive performance estimates can be uncertain when sample sizes are limited [40]. However, the five repetitions reused the same observations and do not constitute independent experimental replications. Dependence arising from overlapping training and validation samples complicates the estimation of cross-validation uncertainty [41]. Accordingly, the reported standard deviations describe variation across the selected partitions rather than confidence intervals for performance in a wider population. Because the adapted baseline was not evaluated using the four additional grouped partitions, its fixed-partition results should not be directly compared with the repeated-validation means of the primary workflows.

Cross-year testing provided an additional assessment of temporal transferability. Research on ecological model evaluation emphasizes that accuracy within one dataset does not necessarily imply reliable transfer to other locations or periods [42]. The mean-SPAD SAVI-RF model achieved R^2^ values of 0.655 and 0.611 in the two testing directions, supporting its predictive potential across the two observed seasons. Nevertheless, both years involved the same site, cultivar, and physical plots. These tests therefore assess transfer between seasons within the existing experiment, rather than independent spatial or cultivar-level generalization. More generally, prediction reliability depends on whether new observations fall within conditions represented by the training data [43]. The applicability of the current models to other environments remains to be established.

Model selection and residual behavior impose further limits on interpretation. Selecting the best-performing configurations from the same out-of-fold results used for comparison can introduce selection-induced optimism [27], even when each candidate workflow uses nested validation. In addition, residual diagnostics for the mean-SPAD SAVI-RF model showed average overestimation in the lowest measured-SPAD quartile and underestimation in the highest quartile (Figure S1). These patterns describe errors conditional on measured SPAD and should not be interpreted as evidence of a particular physiological mechanism. They nevertheless identify parts of the observed range that warrant closer evaluation in future datasets.

This study was restricted to one cultivar, one site, and the flowering stage, and leaf selection was based on relative canopy position rather than a standardized leaf rank or quantitative height threshold. Future work should prioritize independent observations across sites, cultivars, and growth stages; reproducible leaf-position criteria; and complete spectral acquisition and preprocessing records. Concurrent measurements of leaf area, water status, canopy structure, and extracted chlorophyll would help distinguish physiological variation from optical and averaging effects. Within these limits, the study provides evidence for comparing vertically defined SPAD targets and selecting spectral workflows under a consistent evaluation framework.

## 5. Conclusions

Using observations collected during the flowering stage of soybean over two growing seasons, this study combined fractional-order differentiation, spectral indices, and machine learning to compare the spectral associations and predictive performance of upper-layer, lower-layer, and mean SPAD. The main conclusions are as follows:

(1) The maximum association strengths and optimal differentiation orders differed among SPAD targets. In the full-data correlation analysis, both DI and SAVI achieved their maximum |r| values for upper-layer SPAD at an order of 0.4, with values of 0.732 and 0.737, respectively, and optimal wavelength combinations located within approximately 1525–1578 nm. Lower-layer and mean SPAD exhibited their strongest associations at an order of 1.2 using the wavelength pair of 691 and 1578 nm, with maximum |r| values of approximately 0.733 and 0.810, respectively. These results characterize associations within the current dataset and do not directly indicate model generalization performance.
(2) Under the original fixed plot-grouped five-fold validation scheme, RF achieved the highest validation R^2^ for upper-layer SPAD across all three primary input feature sets, whereas BPNN performed best for lower-layer SPAD. Mean SPAD showed greater predictability when using the same input feature set and algorithm. Both DI-J-RF and SAVI-J-RF achieved a validation R^2^ of 0.636 and an RMSE of 2.450. SAVI-J-RF achieved an R^2^ of 0.597 ± 0.064 (mean ± standard deviation) across five validation repetitions, with cross-year test R^2^ values of 0.655 and 0.611 in the two directions. These findings indicate that its favorable predictive performance was not restricted to the original fixed partition.
(3) Neither ZU nor NDI demonstrated a consistent predictive advantage. Comparison with the literature-adapted baseline showed no consistent advantage of the proposed workflows across SPAD targets and algorithms. The current results support the equal-weight mean of upper- and lower-layer SPAD as a relatively predictable estimation target under the present experimental conditions, but they do not establish that it better represents whole-plant chlorophyll status. These conclusions are limited to the cultivar, site, and flowering-stage conditions investigated, and their applicability requires evaluation using independent external data.

Overall, this study identified differences in spectral associations and predictive performance among SPAD targets at different vertical canopy positions. The equal-weight mean of upper- and lower-layer SPAD showed favorable predictability within the current experiment. These findings provide empirical evidence to inform the integration of sampling information and the selection of modeling targets for hyperspectral monitoring of flowering-stage soybean.

## Figures and Tables

**Table 1:** The statistical characteristics of SPAD values measured at different canopy layers during the soybean flowering stage.

Statistical Indicators	Upper-Layer SPAD	Lower-Layer SPAD	Mean SPAD
Sample Size	120	120	120
Maximum Value	57.89	55.74	56.82
Minimum Value	38.00	31.76	35.96
Mean Value	48.72	47.83	48.27
Standard Deviation	4.25	5.03	4.08
Coefficient of Variation/%	8.71	10.53	8.45

Note: Mean SPAD is the arithmetic mean of upper- and lower-layer SPAD values measured on the same plant. The standard deviation was calculated as the sample standard deviation (with a denominator of *n* − 1), and the coefficient of variation was calculated as the standard deviation divided by the mean and multiplied by 100%. All statistics were calculated using the original, unrounded values. The 120 plant-level observations were obtained from 20 physical plots.

**Table 2:** Spectral indices and their corresponding formulas.

Select Index	Computing Formula	Reference
DI	*R_i_* − *R_j_*	[1]
SAVI	(1 + 0.16)(*R_i_* − *R_j_*)/(*R_i_* + *R_j_* + 0.16)	[13] *
NDI	(*R_i_* − *R_j_*)/(*R_i_* + *R_j_*)	Supplementary comparison

Note: *R_i_* and *R_j_* denote the spectral values at the two selected wavelengths. For fractional-order differentiated configurations, the corresponding differentiated spectral values were used. NDI was included in this study as a supplementary comparator and was not restricted to the conventional red and near-infrared wavelength combination used in NDVI. * SAVI was originally proposed by Huete [13], while the parameterization used here followed the implementation adopted for soybean spectral analysis [5].

**Table 3:** Optimal spectral index combinations for upper-canopy SPAD based on fractional-order differentiation.

Spectral Index	Differential Order	Wavelength Position	Correlation	Optimal Order	Spectral Index	Differential Order	Wavelength Position	Correlation	Optimal Order
DI-S	0.2	1560, 1578	0.710	0.4	SAVI-S	0.2	1560, 1578	0.717	0.4
	0.4	1525, 1578	0.732	0.8		0.4	1525, 1577	0.737	0.2
	0.6	1112, 1463	0.707	1.6		0.6	1112, 1463	0.711	0.6
	0.8	1340, 1563	0.714	0.2		0.8	1340, 1563	0.711	0.8
	1.0	1151, 1157	0.700	0.6		1.0	1151, 1157	0.700	1.6
	1.2	1151, 1157	0.692			1.2	1151, 1157	0.693	
	1.4	481, 747	0.700			1.4	481, 747	0.699	
	1.6	706, 1566	0.710			1.6	706, 1566	0.709	
	1.8	594, 702	0.692			1.8	594, 702	0.692	
	2.0	594, 701	0.682			2.0	594, 701	0.682	

Note: DI-S and SAVI-S denote spectral indices developed for upper-canopy SPAD.

**Table 4:** Optimal spectral index combinations for lower-layer SPAD based on fractional-order differentiation.

Spectral Index	Differential Order	Wavelength Position	Correlation	Optimal Order	Spectral Index	Differential Order	Wavelength Position	Correlation	Optimal Order
DI-X	0.2	1154, 1202	0.721	1.2	SAVI-X	0.2	1154, 1202	0.725	1.2
	0.4	1334, 1577	0.720	0.6		0.4	1334, 1577	0.719	0.6
	0.6	1307, 1578	0.730	1.0		0.6	1307, 1578	0.733	1.0
	0.8	1307, 1578	0.723	1.4		0.8	1307, 1578	0.722	0.2
	1.0	519, 915	0.728	0.8		1.0	519, 915	0.729	1.4
	1.2	691, 1578	0.733			1.2	691, 1578	0.733	
	1.4	559, 621	0.723			1.4	559, 621	0.723	
	1.6	654, 691	0.706			1.6	654, 691	0.706	
	1.8	975, 1340	0.700			1.8	975, 1340	0.700	
	2.0	975, 1340	0.696			2.0	975, 1340	0.695	

Note: DI-X and SAVI-X denote spectral indices developed for lower-canopy SPAD.

**Table 5:** Optimal spectral index combinations for mean SPAD based on fractional-order differentiation.

Spectral Index	Differential Order	Wavelength Position	Correlation	Optimal Order	Spectral Index	Differential Order	Wavelength Position	Correlation	Optimal Order
DI-J	0.2	1154, 1203	0.796	1.2	SAVI-J	0.2	1560, 1578	0.794	1.2
	0.4	1525, 1578	0.804	0.6		0.4	1525, 1578	0.807	0.4
	0.6	921, 1272	0.807	0.4		0.6	921, 1272	0.805	0.6
	0.8	1239, 1340	0.802	0.8		0.8	1299, 1564	0.802	0.8
	1	525, 915	0.800	1.0		1	525, 915	0.802	1.0
	1.2	691, 1578	0.810			1.2	691, 1578	0.810	
	1.4	696, 1578	0.788			1.4	696, 1578	0.788	
	1.6	692, 1566	0.785			1.6	692, 1566	0.785	
	1.8	594, 702	0.788			1.8	594, 702	0.788	
	2	594, 701	0.775			2	594, 701	0.775	

Note: DI-J and SAVI-J denote spectral indices developed for mean SPAD.

**Table 6:** Training and out-of-fold validation performance of upper-layer SPAD estimation models.

Comb.	Metric	BPNN		SVR		RF	
		Tr.	Val.	Tr.	Val.	Tr.	Val.
DI-S	R^2^	0.575	0.410	0.549	0.350	0.724	0.457
	RMSE	2.741	3.246	2.824	3.408	2.206	3.116
	NRMSE (%)	5.627	6.664	5.798	6.995	4.529	6.396
	MRE (%)	4.293	4.988	4.118	5.340	3.388	4.817
SAVI-S	R^2^	0.570	0.424	0.555	0.345	0.726	0.461
	RMSE	2.759	3.209	2.804	3.421	2.200	3.105
	NRMSE (%)	5.663	6.587	5.756	7.023	4.518	6.373
	MRE (%)	4.372	4.811	4.077	5.132	3.402	4.838
ZU-S	R^2^	0.552	0.384	0.552	0.361	0.742	0.459
	RMSE	2.809	3.318	2.811	3.380	2.129	3.111
	NRMSE (%)	5.767	6.812	5.769	6.938	4.369	6.385
	MRE (%)	4.415	5.007	4.096	5.072	3.275	4.860
NDI-S	R^2^	0.584	0.380	0.540	0.260	0.723	0.373
	RMSE	2.712	3.329	2.847	3.638	2.203	3.348
	NRMSE (%)	5.567	6.833	5.845	7.467	4.523	6.873
	MRE (%)	4.248	5.298	4.302	5.977	3.451	5.410

Note: S, X, and J denote upper-layer, lower-layer, and mean SPAD, respectively. Training metrics are the arithmetic means of the corresponding metrics from the five outer training folds. Validation metrics were calculated from the pooled 120 unique out-of-fold predictions obtained through five-fold cross-validation grouped by fixed plot.

**Table 7:** Training and out-of-fold validation performance of lower-layer SPAD estimation models.

Comb.	Metric	BPNN		SVR		RF	
		Tr.	Val.	Tr.	Val.	Tr.	Val.
DI-X	R^2^	0.585	0.483	0.559	0.466	0.702	0.474
	RMSE	3.223	3.604	3.316	3.665	2.721	3.635
	NRMSE (%)	6.741	7.535	6.937	7.663	5.691	7.601
	MRE (%)	4.740	5.393	4.453	5.164	3.812	5.175
SAVI-X	R^2^	0.575	0.510	0.554	0.460	0.711	0.487
	RMSE	3.261	3.509	3.336	3.683	2.676	3.591
	NRMSE (%)	6.821	7.337	6.978	7.700	5.597	7.508
	MRE (%)	4.794	5.209	4.394	5.242	3.758	5.113
ZU-X	R^2^	0.579	0.504	0.551	0.464	0.704	0.475
	RMSE	3.245	3.530	3.346	3.669	2.713	3.632
	NRMSE (%)	6.786	7.381	6.999	7.672	5.674	7.595
	MRE (%)	4.730	5.301	4.481	5.367	3.813	5.189
NDI-X	R^2^	0.564	0.386	0.528	0.368	0.705	0.478
	RMSE	3.303	3.927	3.429	3.987	2.709	3.624
	NRMSE (%)	6.908	8.211	7.173	8.336	5.666	7.577
	MRE (%)	5.224	6.219	5.273	6.589	4.000	5.463

**Table 8:** Training and out-of-fold validation performance of mean SPAD estimation models.

Comb.	Metric	BPNN		SVR		RF	
		Tr.	Val.	Tr.	Val.	Tr.	Val.
DI-J	R^2^	0.704	0.617	0.695	0.583	0.820	0.636
	RMSE	2.201	2.515	2.230	2.622	1.708	2.450
	NRMSE (%)	4.561	5.209	4.621	5.431	3.540	5.076
	MRE (%)	3.455	3.881	3.331	4.068	2.682	3.810
SAVI-J	R^2^	0.713	0.627	0.704	0.604	0.829	0.636
	RMSE	2.165	2.479	2.199	2.557	1.662	2.450
	NRMSE (%)	4.487	5.136	4.556	5.298	3.444	5.076
	MRE (%)	3.413	3.773	3.257	3.884	2.599	3.894
ZU-J	R^2^	0.698	0.622	0.707	0.569	0.829	0.620
	RMSE	2.225	2.496	2.189	2.667	1.666	2.505
	NRMSE (%)	4.609	5.171	4.536	5.524	3.452	5.189
	MRE (%)	3.534	3.871	3.301	4.133	2.598	3.976
NDI-J	R^2^	0.714	0.600	0.665	0.500	0.833	0.611
	RMSE	2.162	2.568	2.332	2.873	1.647	2.532
	NRMSE (%)	4.480	5.321	4.833	5.952	3.414	5.246
	MRE (%)	3.622	4.374	3.756	4.901	2.620	4.148

**Table 9:** Repeated plot-grouped validation and cross-year testing results for the primary models.

Model	Repeated Grouped Five-Fold Validation: R^2^		Train 2022 → Test 2023		Train 2023 → Test 2022	
	R^2^	RMSE	R^2^	RMSE	R^2^	RMSE
DI-S-RF	0.435 ± 0.025	3.177 ± 0.071	0.176	3.831	0.445	3.155
SAVI-S-RF	0.442 ± 0.038	3.157 ± 0.105	0.189	3.802	0.455	3.125
ZU-S-RF	0.444 ± 0.045	3.150 ± 0.126	0.199	3.779	0.496	3.006
DI-X-BPNN	0.430 ± 0.081	3.778 ± 0.260	0.504	3.375	0.276	4.364
SAVI-X-BPNN	0.448 ± 0.067	3.721 ± 0.222	0.552	3.207	0.279	4.353
ZU-X-BPNN	0.406 ± 0.088	3.855 ± 0.283	0.573	3.130	0.303	4.281
DI-J-RF	0.589 ± 0.074	2.598 ± 0.225	0.648	2.301	0.601	2.657
SAVI-J-RF	0.597 ± 0.064	2.571 ± 0.198	0.655	2.276	0.611	2.624
ZU-J-BPNN	0.550 ± 0.065	2.719 ± 0.195	0.641	2.322	0.599	2.664

Note: Model configurations correspond to those in Figure 5. Index types and algorithms were held constant across repeated validation and cross-year testing, while feature selection and hyperparameter tuning were repeated within each training dataset. Repeated validation results are reported as the mean ± standard deviation across five repetitions of plot-grouped five-fold validation, each pooling 120 out-of-fold predictions. Each cross-year test used 60 training observations and 60 test observations. The same fixed plots were used in both years. RMSE is expressed in SPAD units.

## Data Availability

The original contributions presented in this study are included in the article/Supplementary Material. Further inquiries can be directed to the corresponding author.

## Supplementary Materials

The following supporting information can be downloaded at: https://www.mdpi.com/article/10.3390/plants15192998/s1.

## Abbreviations

The following abbreviations are used in this manuscript:

BPNN	Backpropagation neural network
CK	Rainfed control
DI	Difference index
FAO	Food and Agriculture Organization of the United Nations
G–L	Grünwald–Letnikov
LiDAR	Light detection and ranging
mNDI	Modified normalized difference index
MRE	Mean relative error
mSR	Modified simple ratio
NDI	Normalized difference index
NDVI	Normalized difference vegetation index
NRMSE	Normalized root mean square error
OOF	Out-of-fold
RF	Random forest
RI	Ratio index
RMSE	Root mean square error
SAVI	Soil-adjusted vegetation index
SD	Standard deviation
SPAD	Soil Plant Analysis Development
SVR	Support vector regression
TVI	Triangular vegetation index
ZU	Combined feature set of DI and SAVI
