# Supplementary material for: Retrieval of Soybean SPAD at Different Vertical Canopy Scales Based on Fractional-Order Derivatives and Spectral Indices
Source: Plants (Basel). 2026 Sep 30;15(19):2998. doi: 10.3390/plants15192998 (PMC13666852; doi:10.3390/plants15192998)
Supplement: Supplementary file 1 [file plants-15-02998-s001.zip › plants-4566358-supplementary.pdf]

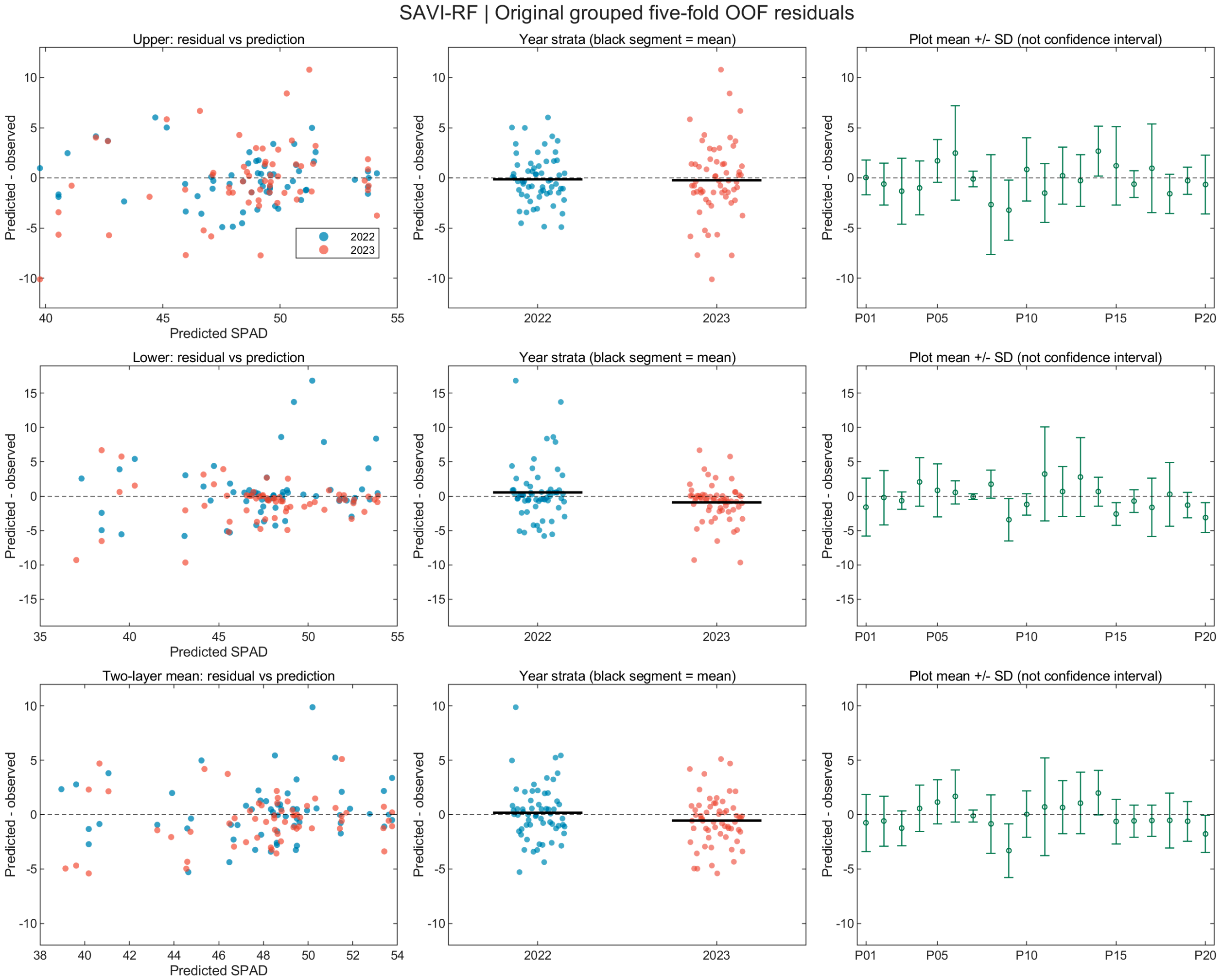

Fig. S1. Out-of-fold residual diagnostics for SAVI-RF under the original fixed plot-grouped five-fold validation. Rows represent upper-canopy, lower-canopy, and two-layer mean SPAD, respectively. Columns show residuals versus predicted values, residual distributions by year, and plot mean residuals with standard deviations. Blue denotes 2022 and red denotes 2023. Horizontal dashed lines indicate zero residual; short black lines indicate mean residuals by year. Each plot contributes six observations across the two years. Error bars denote standard deviations, not confidence intervals. SAVI-RF is shown consistently for comparisons among layers; this does not imply that RF was optimal for every layer. Upper, Lower, and Two-layer mean in the figure correspond to these three targets. Measured-SPAD quartile diagnostics are provided in Table S10.

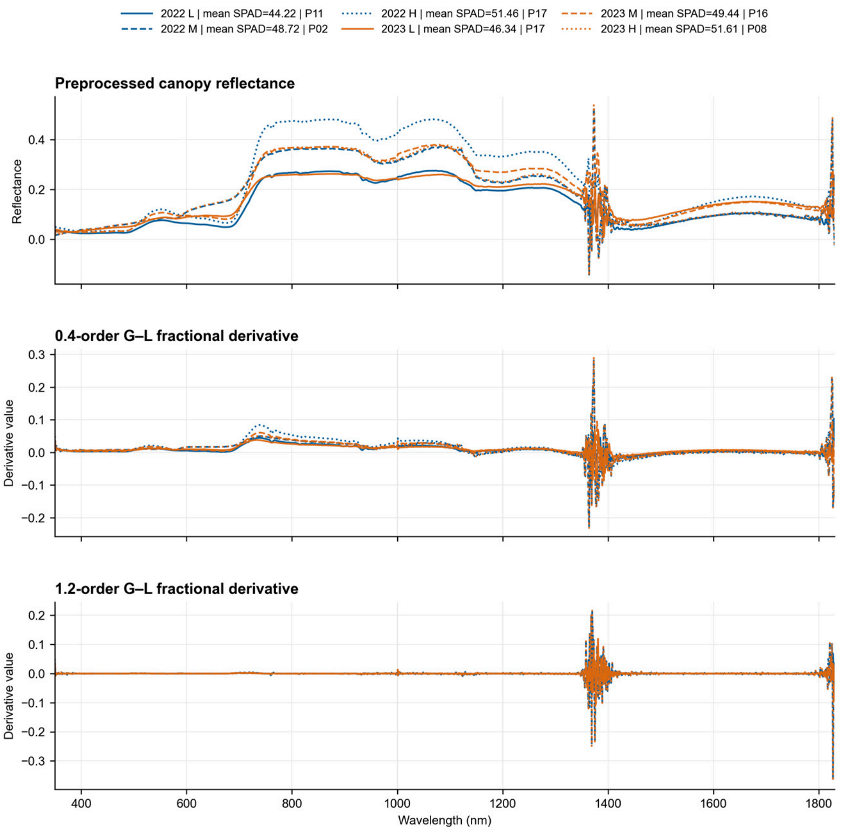

Six observations were selected from the actual 120 plant-level records: low, medium, and high mean-SPAD strata within each year. Selection used the observation nearest the stratum median; no correlation or model-performance criterion was used.

Fig.S2. Representative canopy spectral curves selected from the 120 paired plant-level observations. Within each year, observations were divided into low, medium, and high strata according to the measured mean SPAD, and the observation nearest the median of each stratum was selected. The same six observations are shown for preprocessed canopy reflectance, 0.4-order G–L fractional-order differentiated spectra, and 1.2-order G–L fractional-order differentiated spectra. Line colors distinguish years, and line styles distinguish within-year mean-SPAD strata. The curves are provided to illustrate observed spectral characteristics and are not model predictions or evidence of independent physiological responses at individual wavelengths.

Table S1. Seven candidate index types for the literature-adapted baseline

Index	Implemented formula	Fixed parameters
RI	R_i / R_j	None
DI	$Ri - R_j$	None
mSR	$(R_i - R455) / (R_j - R455)$	455 nm
mNDI	$(R_i - R_j) / (R_i + R_j - 2R455)$	455 nm
TVI	$0.5 \times [120(R_i - R550) - 200(R_j - R550)]$	550 nm
SAVI	$1.16 \times (R_i - R_j) / (R_i + R_j + 0.16)$	L=0.16
NDVI	$(R_i - R_j) / (R_i + R_j)$	Baseline terminology retained; candidate wavelengths are not restricted to red and near-infrared bands

Baseline formulas follow [5]

Table S2. DI, SAVI, and ZU metrics under the original fixed five-fold and cross-year tests, including undifferentiated smoothed spectra

Family	Target	Algorithm	N	R²	RMSE	NRMSE_percent	MRE_percent	Bias
DI	lower	BPNN	120	0.483345	3.603544	7.534723	5.393498	0.085861
DI	lower	RF	120	0.474216	3.635242	7.601001	5.175159	-0.203162
DI	lower	SVR	120	0.465534	3.665132	7.663499	5.163682	0.495690
DI	mean	BPNN	120	0.616756	2.514501	5.209142	3.881497	0.060373
DI	mean	RF	120	0.636044	2.450410	5.076368	3.809618	-0.081652
DI	mean	SVR	120	0.583364	2.621758	5.431340	4.068111	0.126528
DI	upper	BPNN	120	0.410371	3.246298	6.663718	4.987981	-0.055612
DI	upper	RF	120	0.456752	3.116006	6.396264	4.817165	-0.116470
DI	upper	SVR	120	0.350207	3.407899	6.995438	5.339998	-0.219499
SAVI	lower	BPNN	120	0.510061	3.509139	7.337331	5.209128	0.095051
SAVI	lower	RF	120	0.486939	3.590987	7.508467	5.113438	-0.181093
SAVI	lower	SVR	120	0.460382	3.682752	7.700342	5.241932	0.611028
SAVI	mean	BPNN	120	0.627421	2.479268	5.136151	3.772539	0.040743
SAVI	mean	RF	120	0.636052	2.450381	5.076308	3.893904	-0.197951
SAVI	mean	SVR	120	0.603580	2.557359	5.297929	3.884407	0.125842
SAVI	upper	BPNN	120	0.423893	3.208861	6.586871	4.810681	0.030671
SAVI	upper	RF	120	0.460689	3.104695	6.373048	4.837522	-0.190335
SAVI	upper	SVR	120	0.345113	3.421232	7.022807	5.132006	-0.175713
ZU	lower	BPNN	120	0.504248	3.529893	7.380726	5.300659	0.096891
ZU	lower	RF	120	0.475055	3.632338	7.594930	5.188725	-0.100904
ZU	lower	SVR	120	0.464384	3.669072	7.671738	5.367438	0.664495
ZU	mean	BPNN	120	0.622314	2.496202	5.171234	3.870675	0.049814
ZU	mean	RF	120	0.619763	2.504618	5.188669	3.976398	-0.159677
ZU	mean	SVR	120	0.568961	2.666690	5.524423	4.132851	0.170690
ZU	upper	BPNN	120	0.383876	3.318433	6.811790	5.007104	0.083619
ZU	upper	RF	120	0.458626	3.110627	6.385223	4.859580	-0.104727
ZU	upper	SVR	120	0.360767	3.380095	6.938363	5.072269	-0.006782
seven_indices	lower	BPNN	120	0.466018	3.663470	7.660023	5.597501	-0.090111
seven_indices	lower	RF	120	0.466827	3.660693	7.654218	5.463235	-0.312938
seven_indices	lower	SVR	120	0.469399	3.651854	7.635737	5.221968	0.342385
seven_indices	mean	BPNN	120	0.629321	2.472937	5.123037	3.917065	-0.045158
seven_indices	mean	RF	120	0.669978	2.333380	4.833925	3.714695	-0.009199
seven_indices	mean	SVR	120	0.590836	2.598142	5.382416	4.112350	0.091774
seven_indices	upper	BPNN	120	0.492113	3.012886	6.184589	4.708049	-0.036360
seven_indices	upper	RF	120	0.486265	3.030182	6.220093	4.906442	-0.159300
seven_indices	upper	SVR	120	0.353892	3.398222	6.975574	5.264585	-0.063980
DI	lower	BPNN	120	0.450948	3.714806	7.767363	5.631289	-0.064916
DI	lower	RF	120	0.496187	3.558476	7.440490	5.121232	-0.031221
DI	lower	SVR	120	0.399112	3.886209	8.125754	5.591619	0.582867
DI	mean	BPNN	120	0.493357	2.891114	5.989348	4.670858	-0.104922
DI	mean	RF	120	0.579648	2.633423	5.455505	4.332580	-0.075726
DI	mean	SVR	120	0.559360	2.696224	5.585608	4.218632	0.155356
DI	upper	BPNN	120	0.323132	3.478172	7.139689	5.384648	-0.259424
DI	upper	RF	120	0.419594	3.220811	6.611401	5.082945	-0.210777
DI	upper	SVR	120	0.316332	3.495601	7.175466	5.246255	0.021816
SAVI	lower	BPNN	120	0.417896	3.824987	7.997742	5.623014	-0.045576

SAVI	lower	RF	120	0.407121	3.860225	8.071423	5.587734	-0.022240
SAVI	lower	SVR	120	0.367741	3.986365	8.335171	5.585135	0.892035
SAVI	mean	BPNN	120	0.547193	2.733197	5.662202	4.380521	-0.179960
SAVI	mean	RF	120	0.591947	2.594612	5.375103	4.160508	-0.161165
SAVI	mean	SVR	120	0.548556	2.729078	5.653668	4.277538	0.389445
SAVI	upper	BPNN	120	0.323757	3.476566	7.136391	5.587284	-0.243502
SAVI	upper	RF	120	0.329872	3.460812	7.104053	5.518038	-0.262284
SAVI	upper	SVR	120	0.252925	3.654108	7.500835	5.688019	0.057499
ZU	lower	BPNN	120	0.438911	3.755304	7.852043	5.810049	-0.242247
ZU	lower	RF	120	0.474297	3.634962	7.600416	5.364472	-0.044888
ZU	lower	SVR	120	0.385593	3.929683	8.216655	5.826774	0.419267
ZU	mean	BPNN	120	0.558458	2.698983	5.591322	4.317078	-0.136134
ZU	mean	RF	120	0.570237	2.662740	5.516240	4.355629	-0.105551
ZU	mean	SVR	120	0.567015	2.672702	5.536878	4.186610	0.458686
ZU	upper	BPNN	120	0.335520	3.446198	7.074056	5.476277	-0.188650
ZU	upper	RF	120	0.434703	3.178612	6.524777	5.032654	-0.220589
ZU	upper	SVR	120	0.311680	3.507473	7.199835	5.261907	0.034555
DI	lower	BPNN	60	0.503704	3.374605	6.951747	5.784887	-1.390016
DI	lower	RF	60	0.525353	3.300181	6.798432	5.184812	-1.416691
DI	lower	SVR	60	0.537629	3.257227	6.709945	5.252637	-0.577708
DI	mean	BPNN	60	0.629442	2.359193	4.848803	3.866220	-0.725811
DI	mean	RF	60	0.647637	2.300545	4.728265	3.742895	-0.804199
DI	mean	SVR	60	0.671292	2.221979	4.566791	3.557634	-0.280028
DI	upper	BPNN	60	0.198612	3.778872	7.748823	5.765083	-0.073250
DI	upper	RF	60	0.176299	3.831117	7.855955	5.813229	-0.090368
DI	upper	SVR	60	0.191257	3.796172	7.784299	5.591328	0.238938
SAVI	lower	BPNN	60	0.551875	3.206655	6.605768	5.285794	-1.424136
SAVI	lower	RF	60	0.535068	3.266234	6.728500	5.037833	-1.431270
SAVI	lower	SVR	60	0.555437	3.193883	6.579455	5.094537	-0.645151
SAVI	mean	BPNN	60	0.654331	2.278585	4.683132	3.687052	-0.744716
SAVI	mean	RF	60	0.655075	2.276133	4.678091	3.654922	-0.758563
SAVI	mean	SVR	60	0.610844	2.417670	4.968990	3.735006	-0.588939
SAVI	upper	BPNN	60	0.213933	3.742576	7.674397	5.689722	-0.102768
SAVI	upper	RF	60	0.188628	3.802339	7.796944	5.740712	-0.083686
SAVI	upper	SVR	60	0.204824	3.764198	7.718734	5.571099	0.333045
ZU	lower	BPNN	60	0.573129	3.129687	6.447211	4.958720	-1.403488
ZU	lower	RF	60	0.525930	3.298177	6.794303	4.990333	-1.384156
ZU	lower	SVR	60	0.550667	3.210972	6.614660	4.961299	-0.489232
ZU	mean	BPNN	60	0.641086	2.321829	4.772011	3.824170	-0.736944
ZU	mean	RF	60	0.643487	2.314052	4.756026	3.716290	-0.788743
ZU	mean	SVR	60	0.676966	2.202721	4.527210	3.569976	-0.215929
ZU	upper	BPNN	60	0.185814	3.808927	7.810452	5.973517	0.105858
ZU	upper	RF	60	0.198684	3.778701	7.748473	5.736459	-0.079195
ZU	upper	SVR	60	0.229734	3.704769	7.596871	5.589763	0.218014
seven_indices	lower	BPNN	60	0.512597	3.344233	6.889180	5.431839	-1.391525
seven_indices	lower	RF	60	0.482438	3.446147	7.099124	5.292492	-1.526911
seven_indices	lower	SVR	60	0.439104	3.587514	7.390344	5.820365	-1.068576
seven_indices	mean	BPNN	60	0.673809	2.213457	4.549275	3.607115	-0.778425
seven_indices	mean	RF	60	0.658166	2.265910	4.657082	3.568650	-0.799799

seven_indices	mean	SVR	60	0.650604	2.290836	4.708311	3.517771	-0.635356
seven_indices	upper	BPNN	60	0.249203	3.657650	7.500249	5.563909	-0.019089
seven_indices	upper	RF	60	0.205593	3.762376	7.714998	5.610375	-0.074904
seven_indices	upper	SVR	60	0.157368	3.874892	7.945719	5.680413	-0.367981
DI	lower	BPNN	60	0.502188	3.379755	6.962355	5.607790	-1.392548
DI	lower	RF	60	0.508269	3.359048	6.919700	5.247886	-1.395679
DI	lower	SVR	60	0.475553	3.468991	7.146183	5.513007	-0.576045
DI	mean	BPNN	60	0.624141	2.376008	4.883363	3.946183	-0.670595
DI	mean	RF	60	0.651278	2.288625	4.703767	3.769622	-0.750159
DI	mean	SVR	60	0.608667	2.424424	4.982871	3.750966	-0.320853
DI	upper	BPNN	60	0.174104	3.836219	7.866418	5.850854	0.093240
DI	upper	RF	60	0.178921	3.825017	7.843446	5.701064	-0.077484
DI	upper	SVR	60	0.170457	3.844679	7.883765	5.743789	0.182207
SAVI	lower	BPNN	60	0.484698	3.438613	7.083604	5.642970	-1.414880
SAVI	lower	RF	60	0.447293	3.561231	7.336201	5.549190	-1.357057
SAVI	lower	SVR	60	0.517024	3.329011	6.857823	4.865297	-0.150928
SAVI	mean	BPNN	60	0.600248	2.450365	5.036188	4.143039	-0.733749
SAVI	mean	RF	60	0.643800	2.313034	4.753933	3.760959	-0.715072
SAVI	mean	SVR	60	0.599341	2.453143	5.041897	3.840791	-0.451993
SAVI	upper	BPNN	60	0.096152	4.013178	8.229282	6.151752	-0.115320
SAVI	upper	RF	60	0.157058	3.875606	7.947182	5.730169	-0.097587
SAVI	upper	SVR	60	0.052354	4.109261	8.426308	6.052848	-0.131649
ZU	lower	BPNN	60	0.502188	3.379755	6.962355	5.607790	-1.392548
ZU	lower	RF	60	0.508269	3.359048	6.919700	5.247886	-1.395679
ZU	lower	SVR	60	0.475553	3.468991	7.146183	5.513007	-0.576045
ZU	mean	BPNN	60	0.656844	2.270290	4.666083	3.741388	-0.666037
ZU	mean	RF	60	0.640807	2.322733	4.773868	3.787694	-0.741792
ZU	mean	SVR	60	0.602467	2.443552	5.022185	3.757534	-0.428669
ZU	upper	BPNN	60	0.174104	3.836219	7.866418	5.850854	0.093240
ZU	upper	RF	60	0.178921	3.825017	7.843446	5.701064	-0.077484
ZU	upper	SVR	60	0.170457	3.844679	7.883765	5.743789	0.182207
DI	lower	BPNN	60	0.275530	4.364405	9.264603	6.242239	1.574511
DI	lower	RF	60	0.305675	4.272639	9.069804	6.117572	1.460047
DI	lower	SVR	60	0.184413	4.630736	9.829960	6.849915	1.964155
DI	mean	BPNN	60	0.577026	2.734663	5.710696	4.350215	0.822048
DI	mean	RF	60	0.600747	2.656875	5.548253	4.206012	0.793918
DI	mean	SVR	60	0.474752	3.047400	6.363771	4.911846	1.187588
DI	upper	BPNN	60	0.482328	3.045930	6.258975	4.808989	0.094126
DI	upper	RF	60	0.444697	3.154696	6.482474	4.952697	0.036897
DI	upper	SVR	60	0.383465	3.324079	6.830534	5.399998	0.231440
SAVI	lower	BPNN	60	0.279378	4.352799	9.239965	6.223462	1.560621
SAVI	lower	RF	60	0.301777	4.284616	9.095229	6.121966	1.479969
SAVI	lower	SVR	60	0.187156	4.622941	9.813413	6.845813	1.955866
SAVI	mean	BPNN	60	0.569545	2.758743	5.760980	4.421365	0.836127
SAVI	mean	RF	60	0.610663	2.623676	5.478924	4.097419	0.773534
SAVI	mean	SVR	60	0.472421	3.054153	6.377874	4.943851	1.221687
SAVI	upper	BPNN	60	0.486522	3.033565	6.233567	4.746327	0.139609
SAVI	upper	RF	60	0.455222	3.124658	6.420751	4.856866	0.075536
SAVI	upper	SVR	60	0.386399	3.316159	6.814260	5.358395	0.384016

ZU	lower	BPNN	60	0.302907	4.281147	9.087865	6.262636	1.397095
ZU	lower	RF	60	0.292570	4.312771	9.154995	6.326886	1.480360
ZU	lower	SVR	60	0.164270	4.687570	9.950606	6.967700	2.095861
ZU	mean	BPNN	60	0.598519	2.664278	5.563712	4.292575	0.767450
ZU	mean	RF	60	0.594937	2.676139	5.588482	4.228126	0.809375
ZU	mean	SVR	60	0.486820	3.012187	6.290238	4.929675	1.315497
ZU	upper	BPNN	60	0.473642	3.071377	6.311266	4.941536	0.237853
ZU	upper	RF	60	0.495805	3.006020	6.176965	4.630355	0.014820
ZU	upper	SVR	60	0.373323	3.351308	6.886486	5.487374	0.358564
seven_indices	lower	BPNN	60	0.314368	4.245806	9.012845	6.552386	1.389260
seven_indices	lower	RF	60	0.307183	4.267995	9.059948	6.235515	1.438292
seven_indices	lower	SVR	60	0.306921	4.268804	9.061664	6.416070	1.436990
seven_indices	mean	BPNN	60	0.566448	2.768648	5.781665	4.521417	0.945673
seven_indices	mean	RF	60	0.601945	2.652888	5.539927	4.235451	0.734583
seven_indices	mean	SVR	60	0.523478	2.902609	6.061411	4.718380	1.136242
seven_indices	upper	BPNN	60	0.548148	2.845709	5.847548	4.620798	0.106760
seven_indices	upper	RF	60	0.579039	2.746713	5.644124	4.393292	0.080292
seven_indices	upper	SVR	60	0.402470	3.272446	6.724435	5.384646	0.485086
DI	lower	BPNN	60	0.269331	4.383036	9.304153	6.395355	1.503555
DI	lower	RF	60	0.286273	4.331925	9.195655	6.237379	1.459618
DI	lower	SVR	60	0.152339	4.720911	10.021381	7.000133	1.809796
DI	mean	BPNN	60	0.518151	2.918790	6.095201	4.210495	0.745540
DI	mean	RF	60	0.557600	2.796757	5.840364	4.196810	0.747390
DI	mean	SVR	60	0.514816	2.928872	6.116254	4.366285	0.780484
DI	upper	BPNN	60	0.491627	3.018447	6.202501	4.715288	0.137611
DI	upper	RF	60	0.506904	2.972750	6.108599	4.681751	0.037079
DI	upper	SVR	60	0.384753	3.320606	6.823396	5.408679	0.565736
SAVI	lower	BPNN	60	0.263615	4.400147	9.340475	6.256086	1.449495
SAVI	lower	RF	60	0.300752	4.287759	9.101901	6.164851	1.433100
SAVI	lower	SVR	60	0.162810	4.691663	9.959293	6.516006	1.958721
SAVI	mean	BPNN	60	0.447793	3.124627	6.525041	4.885061	1.041449
SAVI	mean	RF	60	0.535044	2.867169	5.987403	4.414200	0.755554
SAVI	mean	SVR	60	0.481290	3.028373	6.324039	4.773390	0.859951
SAVI	upper	BPNN	60	0.508384	2.968283	6.099421	4.603803	0.084819
SAVI	upper	RF	60	0.421352	3.220326	6.617335	4.975605	0.114127
SAVI	upper	SVR	60	0.412408	3.245117	6.668277	5.258747	0.373420
ZU	lower	BPNN	60	0.269331	4.383036	9.304153	6.395355	1.503555
ZU	lower	RF	60	0.257318	4.418920	9.380324	6.427817	1.444887
ZU	lower	SVR	60	0.152339	4.720911	10.021381	7.000133	1.809796
ZU	mean	BPNN	60	0.518151	2.918790	6.095201	4.210495	0.745540
ZU	mean	RF	60	0.557600	2.796757	5.840364	4.196810	0.747390
ZU	mean	SVR	60	0.514816	2.928872	6.116254	4.366285	0.780484
ZU	upper	BPNN	60	0.479214	3.055078	6.277772	4.836811	0.151546
ZU	upper	RF	60	0.506904	2.972750	6.108599	4.681751	0.037079
ZU	upper	SVR	60	0.384753	3.320606	6.823396	5.408679	0.565736

Note: Scope of validation results in Tables S2–S6 and S11–S12 (Display some data)

Table S3. Means, standard deviations, and ranges across five validation repetitions, including undifferentiated smoothed spectra

Spectral processing	Family	Target	Algorithm	Repeats	Mean_R^2	Mean_RMSE	Mean_NRMSE_percent	Mean_MRE_percent	Mean_Bias	SD_R^2	SD_RMSE	SD_NRMSE_percent	SD_MRE_percent	SD_Bias	Min_R^2	Max_R^2	Min_RMSE	Max_RMSE
Fractional-order differentiated spectra	DI	lower	BPNN	5	0.430	3.778	7.899	5.704	0.104	0.081	0.260	0.543	0.306	0.186	0.293	0.483	3.604	4.216
	DI	lower	RF	5	0.407	3.855	8.061	5.639	-0.039	0.064	0.206	0.432	0.388	0.278	0.310	0.474	3.635	4.164
	DI	lower	SVR	5	0.385	3.921	8.199	5.578	0.656	0.097	0.299	0.625	0.394	0.194	0.218	0.466	3.665	4.434
	DI	mean	BPNN	5	0.575	2.637	5.462	4.113	0.087	0.089	0.263	0.544	0.349	0.213	0.418	0.635	2.455	3.098
	DI	mean	RF	5	0.589	2.598	5.382	4.078	0.010	0.074	0.225	0.465	0.264	0.245	0.459	0.636	2.450	2.987
	DI	mean	SVR	5	0.540	2.747	5.691	4.289	0.228	0.073	0.218	0.452	0.270	0.281	0.445	0.630	2.471	3.025
	DI	upper	BPNN	5	0.399	3.275	6.723	5.074	0.017	0.056	0.151	0.310	0.235	0.183	0.332	0.456	3.118	3.455
	DI	upper	RF	5	0.435	3.177	6.521	5.011	-0.011	0.025	0.071	0.146	0.152	0.135	0.402	0.457	3.116	3.268
	DI	upper	SVR	5	0.363	3.372	6.921	5.336	0.094	0.047	0.125	0.256	0.216	0.212	0.308	0.423	3.212	3.516
	SAVI	lower	BPNN	5	0.448	3.721	7.780	5.566	0.094	0.067	0.222	0.464	0.351	0.139	0.346	0.510	3.509	4.056
	SAVI	lower	RF	5	0.419	3.817	7.982	5.560	-0.024	0.068	0.220	0.460	0.414	0.216	0.315	0.487	3.591	4.150
	SAVI	lower	SVR	5	0.420	3.815	7.977	5.471	0.581	0.059	0.191	0.398	0.296	0.124	0.324	0.470	3.650	4.121
	SAVI	mean	BPNN	5	0.572	2.649	5.488	4.126	0.050	0.081	0.241	0.500	0.362	0.225	0.438	0.637	2.448	3.046
	SAVI	mean	RF	5	0.597	2.571	5.327	4.050	-0.042	0.064	0.198	0.410	0.204	0.249	0.493	0.650	2.403	2.891
	SAVI	mean	SVR	5	0.565	2.672	5.536	4.164	0.180	0.072	0.219	0.453	0.313	0.294	0.461	0.650	2.405	2.983
	SAVI	upper	BPNN	5	0.403	3.265	6.702	5.017	0.039	0.044	0.120	0.247	0.208	0.147	0.349	0.452	3.128	3.410
	SAVI	upper	RF	5	0.442	3.157	6.480	4.976	-0.013	0.038	0.105	0.215	0.152	0.205	0.379	0.468	3.084	3.331
	SAVI	upper	SVR	5	0.372	3.348	6.872	5.245	0.099	0.039	0.106	0.217	0.196	0.195	0.326	0.429	3.195	3.471
	ZU	lower	BPNN	5	0.406	3.855	8.060	5.828	0.151	0.088	0.283	0.591	0.479	0.363	0.275	0.504	3.530	4.270
	ZU	lower	RF	5	0.389	3.915	8.186	5.742	-0.040	0.071	0.228	0.477	0.429	0.303	0.307	0.475	3.632	4.175
	ZU	lower	SVR	5	0.360	4.003	8.371	5.797	0.697	0.088	0.276	0.577	0.447	0.221	0.250	0.464	3.669	4.342
	ZU	mean	BPNN	5	0.550	2.719	5.634	4.228	0.040	0.065	0.195	0.405	0.280	0.265	0.451	0.622	2.496	3.010
	ZU	mean	RF	5	0.588	2.602	5.390	4.172	-0.039	0.062	0.189	0.392	0.248	0.215	0.484	0.632	2.463	2.919
	ZU	mean	SVR	5	0.535	2.762	5.723	4.314	0.199	0.070	0.211	0.437	0.296	0.294	0.445	0.632	2.464	3.025
	ZU	upper	BPNN	5	0.402	3.267	6.707	5.003	0.052	0.040	0.111	0.228	0.158	0.158	0.367	0.452	3.131	3.364
	ZU	upper	RF	5	0.444	3.150	6.466	4.945	0.038	0.045	0.126	0.259	0.180	0.159	0.379	0.487	3.028	3.332
	ZU	upper	SVR	5	0.393	3.292	6.758	5.091	0.008	0.042	0.114	0.233	0.174	0.102	0.341	0.436	3.176	3.431
Undifferentiated	DI	lower	BPNN	5	0.364	3.988	8.339	6.111	0.019	0.100	0.310	0.649	0.621	0.230	0.242	0.451	3.715	4.366
	DI	lower	RF	5	0.404	3.861	8.074	5.737	0.059	0.096	0.306	0.640	0.545	0.225	0.254	0.496	3.558	4.329

smoothed spectra	DI	lower	SVR	5	0.331	4.098	8.568	6.043	0.728	0.061	0.188	0.392	0.524	0.251	0.253	0.399	3.886	4.333
	DI	mean	BPNN	5	0.527	2.791	5.782	4.367	0.088	0.047	0.138	0.286	0.270	0.150	0.464	0.573	2.655	2.972
	DI	mean	RF	5	0.579	2.635	5.458	4.219	0.009	0.026	0.080	0.167	0.143	0.104	0.541	0.603	2.559	2.753
	DI	mean	SVR	5	0.530	2.783	5.766	4.256	0.239	0.032	0.095	0.196	0.052	0.113	0.480	0.559	2.696	2.930
	DI	upper	BPNN	5	0.326	3.461	7.104	5.317	-0.028	0.122	0.303	0.623	0.452	0.254	0.120	0.422	3.214	3.967
	DI	upper	RF	5	0.395	3.285	6.743	5.139	-0.055	0.053	0.141	0.290	0.185	0.298	0.308	0.440	3.164	3.516
	DI	upper	SVR	5	0.331	3.452	7.086	5.240	0.035	0.096	0.245	0.502	0.486	0.299	0.191	0.426	3.203	3.803
	SAVI	lower	BPNN	5	0.348	4.039	8.446	6.075	0.032	0.092	0.276	0.577	0.543	0.145	0.193	0.418	3.825	4.503
	SAVI	lower	RF	5	0.308	4.161	8.699	6.179	0.064	0.112	0.331	0.692	0.534	0.252	0.143	0.407	3.860	4.641
	SAVI	lower	SVR	5	0.298	4.193	8.768	5.977	0.853	0.104	0.299	0.625	0.574	0.168	0.115	0.368	3.986	4.716
	SAVI	mean	BPNN	5	0.481	2.925	6.059	4.654	0.033	0.043	0.122	0.252	0.180	0.193	0.436	0.547	2.733	3.051
	SAVI	mean	RF	5	0.522	2.795	5.791	4.349	0.051	0.103	0.289	0.599	0.275	0.228	0.350	0.595	2.586	3.275
	SAVI	mean	SVR	5	0.442	3.030	6.278	4.680	0.425	0.070	0.194	0.403	0.285	0.162	0.364	0.549	2.729	3.239
	SAVI	upper	BPNN	5	0.310	3.508	7.202	5.550	-0.073	0.063	0.161	0.330	0.201	0.250	0.222	0.384	3.318	3.728
	SAVI	upper	RF	5	0.333	3.448	7.078	5.426	-0.085	0.065	0.169	0.348	0.266	0.260	0.258	0.416	3.230	3.642
	SAVI	upper	SVR	5	0.271	3.605	7.400	5.628	0.123	0.073	0.183	0.375	0.385	0.118	0.177	0.368	3.362	3.834
	ZU	lower	BPNN	5	0.349	4.040	8.448	6.153	0.033	0.077	0.240	0.501	0.441	0.281	0.252	0.439	3.755	4.335
	ZU	lower	RF	5	0.367	3.983	8.328	5.958	0.068	0.082	0.257	0.538	0.430	0.215	0.247	0.474	3.635	4.350
	ZU	lower	SVR	5	0.310	4.163	8.703	6.059	0.676	0.056	0.172	0.359	0.441	0.271	0.248	0.386	3.930	4.348
	ZU	mean	BPNN	5	0.529	2.788	5.775	4.412	-0.005	0.022	0.064	0.133	0.063	0.120	0.504	0.558	2.699	2.860
	ZU	mean	RF	5	0.578	2.639	5.466	4.221	0.002	0.029	0.091	0.189	0.153	0.149	0.535	0.612	2.529	2.770
	ZU	mean	SVR	5	0.522	2.804	5.809	4.302	0.283	0.059	0.173	0.359	0.162	0.163	0.445	0.588	2.608	3.027
	ZU	upper	BPNN	5	0.323	3.467	7.117	5.347	-0.056	0.121	0.302	0.619	0.498	0.268	0.126	0.427	3.201	3.953
	ZU	upper	RF	5	0.397	3.279	6.732	5.133	-0.077	0.058	0.157	0.321	0.198	0.306	0.305	0.446	3.146	3.524
	ZU	upper	SVR	5	0.312	3.503	7.190	5.375	0.075	0.082	0.209	0.429	0.400	0.286	0.191	0.415	3.235	3.803

Table S4. Validation metrics for each of the five partitions

Repeat	Spectral processing	Family	Target	Algorithm	N	R^2	RMSE	NRMSE_percent	MRE_percent	Bias
1	Fractional-order differentiated spectra	DI	lower	BPNN	120	0.562	2.688	5.569	4.206	-0.236
1		DI	lower	RF	120	0.581	2.628	5.445	4.175	-0.266
1		DI	lower	SVR	120	0.534	2.771	5.741	4.436	-0.234
1		DI	mean	BPNN	120	0.425	3.206	6.580	5.007	0.000
1		DI	mean	RF	120	0.467	3.086	6.334	4.839	-0.166
1		DI	mean	SVR	120	0.381	3.325	6.825	5.293	-0.013
1		DI	upper	BPNN	120	0.368	3.987	8.336	6.269	-0.413
1		DI	upper	RF	120	0.324	4.123	8.621	6.194	-0.498
1		DI	upper	SVR	120	0.290	4.223	8.831	6.399	0.368
1		SAVI	lower	BPNN	120	0.529	2.787	5.774	4.423	-0.309
1		SAVI	lower	RF	120	0.579	2.636	5.460	4.270	-0.207
1		SAVI	lower	SVR	120	0.528	2.791	5.783	4.423	-0.087
1		SAVI	mean	BPNN	120	0.439	3.165	6.497	4.864	-0.047
1		SAVI	mean	RF	120	0.487	3.028	6.215	4.752	-0.037
1		SAVI	mean	SVR	120	0.432	3.186	6.541	5.044	-0.085
1		SAVI	upper	BPNN	120	0.274	4.272	8.933	6.791	-0.292
1		SAVI	upper	RF	120	0.367	3.987	8.337	6.136	-0.254
1		SAVI	upper	SVR	120	0.253	4.333	9.060	6.870	0.438

1		ZU	lower	BPNN	120	0.567	2.672	5.536	4.061	0.044
1		ZU	lower	RF	120	0.602	2.563	5.309	4.021	0.068
1		ZU	lower	SVR	120	0.517	2.822	5.847	4.254	0.164
1		ZU	mean	BPNN	120	0.422	3.214	6.598	5.001	-0.005
1		ZU	mean	RF	120	0.440	3.164	6.495	5.052	-0.235
1		ZU	mean	SVR	120	0.415	3.235	6.640	5.070	-0.137
1		ZU	upper	BPNN	120	0.339	4.076	8.523	6.168	-0.154
1		ZU	upper	RF	120	0.246	4.352	9.099	6.645	-0.337
1		ZU	upper	SVR	120	0.347	4.051	8.471	5.791	0.595
1		DI	lower	BPNN	120	0.467	2.964	6.141	4.754	-0.086
1		DI	lower	RF	120	0.506	2.853	5.911	4.422	-0.036
1		DI	lower	SVR	120	0.448	3.018	6.252	4.621	0.175
1		DI	mean	BPNN	120	0.348	3.415	7.010	5.404	-0.038
1	Undifferentiated smoothed spectra	DI	mean	RF	120	0.378	3.333	6.842	5.259	-0.050
1		DI	mean	SVR	120	0.318	3.491	7.166	5.366	0.247
1		DI	upper	BPNN	120	0.288	4.230	8.844	6.657	-0.298
1		DI	upper	RF	120	0.358	4.016	8.397	6.167	-0.250
1		DI	upper	SVR	120	0.248	4.348	9.090	6.757	0.360
1		SAVI	lower	BPNN	120	0.504	2.860	5.925	4.401	-0.122

1		SAVI	lower	RF	120	0.597	2.580	5.345	4.063	0.077
1		SAVI	lower	SVR	120	0.445	3.027	6.271	4.484	0.101
1		SAVI	mean	BPNN	120	0.416	3.231	6.633	5.044	0.011
1		SAVI	mean	RF	120	0.446	3.146	6.457	5.042	-0.278
1		SAVI	mean	SVR	120	0.415	3.235	6.640	5.070	-0.137
1		SAVI	upper	BPNN	120	0.562	2.688	5.569	4.206	-0.236
1		SAVI	upper	RF	120	0.581	2.628	5.445	4.175	-0.266
1		SAVI	upper	SVR	120	0.534	2.771	5.741	4.436	-0.234
1		ZU	lower	BPNN	120	0.425	3.206	6.580	5.007	0.000
1		ZU	lower	RF	120	0.467	3.086	6.334	4.839	-0.166
1		ZU	lower	SVR	120	0.381	3.325	6.825	5.293	-0.013
1		ZU	mean	BPNN	120	0.368	3.987	8.336	6.269	-0.413
1		ZU	mean	RF	120	0.324	4.123	8.621	6.194	-0.498
1		ZU	mean	SVR	120	0.290	4.223	8.831	6.399	0.368
1		ZU	upper	BPNN	120	0.529	2.787	5.774	4.423	-0.309
1		ZU	upper	RF	120	0.579	2.636	5.460	4.270	-0.207
1		ZU	upper	SVR	120	0.528	2.791	5.783	4.423	-0.087
2	Fractional-order differentiated spectra	DI	lower	BPNN	120	0.439	3.165	6.497	4.864	-0.047
2		DI	lower	RF	120	0.487	3.028	6.215	4.752	-0.037

2	DI	lower	SVR	120	0.432	3.186	6.541	5.044	-0.085
2	DI	mean	BPNN	120	0.274	4.272	8.933	6.791	-0.292
2	DI	mean	RF	120	0.367	3.987	8.337	6.136	-0.254
2	DI	mean	SVR	120	0.253	4.333	9.060	6.870	0.438
2	DI	upper	BPNN	120	0.567	2.672	5.536	4.061	0.044
2	DI	upper	RF	120	0.602	2.563	5.309	4.021	0.068
2	DI	upper	SVR	120	0.517	2.822	5.847	4.254	0.164
2	SAVI	lower	BPNN	120	0.422	3.214	6.598	5.001	-0.005
2	SAVI	lower	RF	120	0.440	3.164	6.495	5.052	-0.235
2	SAVI	lower	SVR	120	0.415	3.235	6.640	5.070	-0.137
2	SAVI	mean	BPNN	120	0.339	4.076	8.523	6.168	-0.154
2	SAVI	mean	RF	120	0.246	4.352	9.099	6.645	-0.337
2	SAVI	mean	SVR	120	0.347	4.051	8.471	5.791	0.595
2	SAVI	upper	BPNN	120	0.467	2.964	6.141	4.754	-0.086
2	SAVI	upper	RF	120	0.506	2.853	5.911	4.422	-0.036
2	SAVI	upper	SVR	120	0.448	3.018	6.252	4.621	0.175
2	ZU	lower	BPNN	120	0.348	3.415	7.010	5.404	-0.038
2	ZU	lower	RF	120	0.378	3.333	6.842	5.259	-0.050
2	ZU	lower	SVR	120	0.318	3.491	7.166	5.366	0.247

2		ZU	mean	BPNN	120	0.288	4.230	8.844	6.657	-0.298
2		ZU	mean	RF	120	0.358	4.016	8.397	6.167	-0.250
2		ZU	mean	SVR	120	0.248	4.348	9.090	6.757	0.360
2		ZU	upper	BPNN	120	0.504	2.860	5.925	4.401	-0.122
2		ZU	upper	RF	120	0.597	2.580	5.345	4.063	0.077
2		ZU	upper	SVR	120	0.445	3.027	6.271	4.484	0.101
2		DI	lower	BPNN	120	0.416	3.231	6.633	5.044	0.011
2		DI	lower	RF	120	0.446	3.146	6.457	5.042	-0.278
2		DI	lower	SVR	120	0.415	3.235	6.640	5.070	-0.137
2		DI	mean	BPNN	120	0.562	2.688	5.569	4.206	-0.236
2		DI	mean	RF	120	0.581	2.628	5.445	4.175	-0.266
2		DI	mean	SVR	120	0.534	2.771	5.741	4.436	-0.234
2	Undifferentiated smoothed spectra	DI	upper	BPNN	120	0.425	3.206	6.580	5.007	0.000
2		DI	upper	RF	120	0.467	3.086	6.334	4.839	-0.166
2		DI	upper	SVR	120	0.381	3.325	6.825	5.293	-0.013
2		SAVI	lower	BPNN	120	0.368	3.987	8.336	6.269	-0.413
2		SAVI	lower	RF	120	0.324	4.123	8.621	6.194	-0.498
2		SAVI	lower	SVR	120	0.290	4.223	8.831	6.399	0.368
2		SAVI	mean	BPNN	120	0.529	2.787	5.774	4.423	-0.309

2		SAVI	mean	RF	120	0.579	2.636	5.460	4.270	-0.207
2		SAVI	mean	SVR	120	0.528	2.791	5.783	4.423	-0.087
2		SAVI	upper	BPNN	120	0.439	3.165	6.497	4.864	-0.047
2		SAVI	upper	RF	120	0.487	3.028	6.215	4.752	-0.037
2		SAVI	upper	SVR	120	0.432	3.186	6.541	5.044	-0.085
2		ZU	lower	BPNN	120	0.274	4.272	8.933	6.791	-0.292
2		ZU	lower	RF	120	0.367	3.987	8.337	6.136	-0.254
2		ZU	lower	SVR	120	0.253	4.333	9.060	6.870	0.438
2		ZU	mean	BPNN	120	0.567	2.672	5.536	4.061	0.044
2		ZU	mean	RF	120	0.602	2.563	5.309	4.021	0.068
2		ZU	mean	SVR	120	0.517	2.822	5.847	4.254	0.164
2		ZU	upper	BPNN	120	0.422	3.214	6.598	5.001	-0.005
2		ZU	upper	RF	120	0.440	3.164	6.495	5.052	-0.235
2		ZU	upper	SVR	120	0.415	3.235	6.640	5.070	-0.137
3		DI	lower	BPNN	120	0.339	4.076	8.523	6.168	-0.154
3		DI	lower	RF	120	0.246	4.352	9.099	6.645	-0.337
3	Fractional-order differentiated spectra	DI	lower	SVR	120	0.347	4.051	8.471	5.791	0.595
3		DI	mean	BPNN	120	0.467	2.964	6.141	4.754	-0.086
3		DI	mean	RF	120	0.506	2.853	5.911	4.422	-0.036

3	DI	mean	SVR	120	0.448	3.018	6.252	4.621	0.175
3	DI	upper	BPNN	120	0.348	3.415	7.010	5.404	-0.038
3	DI	upper	RF	120	0.378	3.333	6.842	5.259	-0.050
3	DI	upper	SVR	120	0.318	3.491	7.166	5.366	0.247
3	SAVI	lower	BPNN	120	0.288	4.230	8.844	6.657	-0.298
3	SAVI	lower	RF	120	0.358	4.016	8.397	6.167	-0.250
3	SAVI	lower	SVR	120	0.248	4.348	9.090	6.757	0.360
3	SAVI	mean	BPNN	120	0.504	2.860	5.925	4.401	-0.122
3	SAVI	mean	RF	120	0.597	2.580	5.345	4.063	0.077
3	SAVI	mean	SVR	120	0.445	3.027	6.271	4.484	0.101
3	SAVI	upper	BPNN	120	0.416	3.231	6.633	5.044	0.011
3	SAVI	upper	RF	120	0.446	3.146	6.457	5.042	-0.278
3	SAVI	upper	SVR	120	0.415	3.235	6.640	5.070	-0.137
3	ZU	lower	BPNN	120	0.562	2.688	5.569	4.206	-0.236
3	ZU	lower	RF	120	0.581	2.628	5.445	4.175	-0.266
3	ZU	lower	SVR	120	0.534	2.771	5.741	4.436	-0.234
3	ZU	mean	BPNN	120	0.425	3.206	6.580	5.007	0.000
3	ZU	mean	RF	120	0.467	3.086	6.334	4.839	-0.166
3	ZU	mean	SVR	120	0.381	3.325	6.825	5.293	-0.013

3		ZU	upper	BPNN	120	0.368	3.987	8.336	6.269	-0.413
3		ZU	upper	RF	120	0.324	4.123	8.621	6.194	-0.498
3		ZU	upper	SVR	120	0.290	4.223	8.831	6.399	0.368
3		DI	lower	BPNN	120	0.529	2.787	5.774	4.423	-0.309
3		DI	lower	RF	120	0.579	2.636	5.460	4.270	-0.207
3		DI	lower	SVR	120	0.528	2.791	5.783	4.423	-0.087
3		DI	mean	BPNN	120	0.439	3.165	6.497	4.864	-0.047
3		DI	mean	RF	120	0.487	3.028	6.215	4.752	-0.037
3		DI	mean	SVR	120	0.432	3.186	6.541	5.044	-0.085
3		DI	upper	BPNN	120	0.274	4.272	8.933	6.791	-0.292
3	Undifferentiated smoothed spectra	DI	upper	RF	120	0.367	3.987	8.337	6.136	-0.254
3		DI	upper	SVR	120	0.253	4.333	9.060	6.870	0.438
3		SAVI	lower	BPNN	120	0.567	2.672	5.536	4.061	0.044
3		SAVI	lower	RF	120	0.602	2.563	5.309	4.021	0.068
3		SAVI	lower	SVR	120	0.517	2.822	5.847	4.254	0.164
3		SAVI	mean	BPNN	120	0.422	3.214	6.598	5.001	-0.005
3		SAVI	mean	RF	120	0.440	3.164	6.495	5.052	-0.235
3		SAVI	mean	SVR	120	0.415	3.235	6.640	5.070	-0.137
3		SAVI	upper	BPNN	120	0.339	4.076	8.523	6.168	-0.154

3		SAVI	upper	RF	120	0.246	4.352	9.099	6.645	-0.337
3		SAVI	upper	SVR	120	0.347	4.051	8.471	5.791	0.595
3		ZU	lower	BPNN	120	0.467	2.964	6.141	4.754	-0.086
3		ZU	lower	RF	120	0.506	2.853	5.911	4.422	-0.036
3		ZU	lower	SVR	120	0.448	3.018	6.252	4.621	0.175
3		ZU	mean	BPNN	120	0.348	3.415	7.010	5.404	-0.038
3		ZU	mean	RF	120	0.378	3.333	6.842	5.259	-0.050
3		ZU	mean	SVR	120	0.318	3.491	7.166	5.366	0.247
3		ZU	upper	BPNN	120	0.288	4.230	8.844	6.657	-0.298
3		ZU	upper	RF	120	0.358	4.016	8.397	6.167	-0.250
3		ZU	upper	SVR	120	0.248	4.348	9.090	6.757	0.360
4		DI	lower	BPNN	120	0.504	2.860	5.925	4.401	-0.122
4		DI	lower	RF	120	0.597	2.580	5.345	4.063	0.077
4		DI	lower	SVR	120	0.445	3.027	6.271	4.484	0.101
4	Fractional-order differentiated spectra	DI	mean	BPNN	120	0.416	3.231	6.633	5.044	0.011
4		DI	mean	RF	120	0.446	3.146	6.457	5.042	-0.278
4		DI	mean	SVR	120	0.415	3.235	6.640	5.070	-0.137
4		DI	upper	BPNN	120	0.562	2.688	5.569	4.206	-0.236
4		DI	upper	RF	120	0.581	2.628	5.445	4.175	-0.266

4	DI	upper	SVR	120	0.534	2.771	5.741	4.436	-0.234
4	SAVI	lower	BPNN	120	0.425	3.206	6.580	5.007	0.000
4	SAVI	lower	RF	120	0.467	3.086	6.334	4.839	-0.166
4	SAVI	lower	SVR	120	0.381	3.325	6.825	5.293	-0.013
4	SAVI	mean	BPNN	120	0.368	3.987	8.336	6.269	-0.413
4	SAVI	mean	RF	120	0.324	4.123	8.621	6.194	-0.498
4	SAVI	mean	SVR	120	0.290	4.223	8.831	6.399	0.368
4	SAVI	upper	BPNN	120	0.529	2.787	5.774	4.423	-0.309
4	SAVI	upper	RF	120	0.579	2.636	5.460	4.270	-0.207
4	SAVI	upper	SVR	120	0.528	2.791	5.783	4.423	-0.087
4	ZU	lower	BPNN	120	0.439	3.165	6.497	4.864	-0.047
4	ZU	lower	RF	120	0.487	3.028	6.215	4.752	-0.037
4	ZU	lower	SVR	120	0.432	3.186	6.541	5.044	-0.085
4	ZU	mean	BPNN	120	0.274	4.272	8.933	6.791	-0.292
4	ZU	mean	RF	120	0.367	3.987	8.337	6.136	-0.254
4	ZU	mean	SVR	120	0.253	4.333	9.060	6.870	0.438
4	ZU	upper	BPNN	120	0.567	2.672	5.536	4.061	0.044
4	ZU	upper	RF	120	0.602	2.563	5.309	4.021	0.068
4	ZU	upper	SVR	120	0.517	2.822	5.847	4.254	0.164

4		DI	lower	BPNN	120	0.422	3.214	6.598	5.001	-0.005
4		DI	lower	RF	120	0.440	3.164	6.495	5.052	-0.235
4		DI	lower	SVR	120	0.415	3.235	6.640	5.070	-0.137
4		DI	mean	BPNN	120	0.339	4.076	8.523	6.168	-0.154
4		DI	mean	RF	120	0.246	4.352	9.099	6.645	-0.337
4		DI	mean	SVR	120	0.347	4.051	8.471	5.791	0.595
4		DI	upper	BPNN	120	0.467	2.964	6.141	4.754	-0.086
4		DI	upper	RF	120	0.506	2.853	5.911	4.422	-0.036
4		DI	upper	SVR	120	0.448	3.018	6.252	4.621	0.175
4	Undifferentiated smoothed spectra	SAVI	lower	BPNN	120	0.348	3.415	7.010	5.404	-0.038
4		SAVI	lower	RF	120	0.378	3.333	6.842	5.259	-0.050
4		SAVI	lower	SVR	120	0.318	3.491	7.166	5.366	0.247
4		SAVI	mean	BPNN	120	0.288	4.230	8.844	6.657	-0.298
4		SAVI	mean	RF	120	0.358	4.016	8.397	6.167	-0.250
4		SAVI	mean	SVR	120	0.248	4.348	9.090	6.757	0.360
4		SAVI	upper	BPNN	120	0.504	2.860	5.925	4.401	-0.122
4		SAVI	upper	RF	120	0.597	2.580	5.345	4.063	0.077
4		SAVI	upper	SVR	120	0.445	3.027	6.271	4.484	0.101
4		ZU	lower	BPNN	120	0.416	3.231	6.633	5.044	0.011

4		ZU	lower	RF	120	0.446	3.146	6.457	5.042	-0.278
4		ZU	lower	SVR	120	0.415	3.235	6.640	5.070	-0.137
4		ZU	mean	BPNN	120	0.562	2.688	5.569	4.206	-0.236
4		ZU	mean	RF	120	0.581	2.628	5.445	4.175	-0.266
4		ZU	mean	SVR	120	0.534	2.771	5.741	4.436	-0.234
4		ZU	upper	BPNN	120	0.425	3.206	6.580	5.007	0.000
4		ZU	upper	RF	120	0.467	3.086	6.334	4.839	-0.166
4		ZU	upper	SVR	120	0.381	3.325	6.825	5.293	-0.013
5		DI	lower	BPNN	120	0.368	3.987	8.336	6.269	-0.413
5		DI	lower	RF	120	0.324	4.123	8.621	6.194	-0.498
5		DI	lower	SVR	120	0.290	4.223	8.831	6.399	0.368
5		DI	mean	BPNN	120	0.529	2.787	5.774	4.423	-0.309
5		DI	mean	RF	120	0.579	2.636	5.460	4.270	-0.207
5	Fractional-order differentiated spectra	DI	mean	SVR	120	0.528	2.791	5.783	4.423	-0.087
5		DI	upper	BPNN	120	0.439	3.165	6.497	4.864	-0.047
5		DI	upper	RF	120	0.487	3.028	6.215	4.752	-0.037
5		DI	upper	SVR	120	0.432	3.186	6.541	5.044	-0.085
5		SAVI	lower	BPNN	120	0.274	4.272	8.933	6.791	-0.292
5		SAVI	lower	RF	120	0.367	3.987	8.337	6.136	-0.254

5		SAVI	lower	SVR	120	0.253	4.333	9.060	6.870	0.438
5		SAVI	mean	BPNN	120	0.567	2.672	5.536	4.061	0.044
5		SAVI	mean	RF	120	0.602	2.563	5.309	4.021	0.068
5		SAVI	mean	SVR	120	0.517	2.822	5.847	4.254	0.164
5		SAVI	upper	BPNN	120	0.422	3.214	6.598	5.001	-0.005
5		SAVI	upper	RF	120	0.440	3.164	6.495	5.052	-0.235
5		SAVI	upper	SVR	120	0.415	3.235	6.640	5.070	-0.137
5		ZU	lower	BPNN	120	0.339	4.076	8.523	6.168	-0.154
5		ZU	lower	RF	120	0.246	4.352	9.099	6.645	-0.337
5		ZU	lower	SVR	120	0.347	4.051	8.471	5.791	0.595
5		ZU	mean	BPNN	120	0.467	2.964	6.141	4.754	-0.086
5		ZU	mean	RF	120	0.506	2.853	5.911	4.422	-0.036
5		ZU	mean	SVR	120	0.448	3.018	6.252	4.621	0.175
5		ZU	upper	BPNN	120	0.348	3.415	7.010	5.404	-0.038
5		ZU	upper	RF	120	0.378	3.333	6.842	5.259	-0.050
5		ZU	upper	SVR	120	0.318	3.491	7.166	5.366	0.247
5		DI	lower	BPNN	120	0.288	4.230	8.844	6.657	-0.298
5	Undifferentiated smoothed spectra	DI	lower	RF	120	0.358	4.016	8.397	6.167	-0.250
5		DI	lower	SVR	120	0.248	4.348	9.090	6.757	0.360

5	DI	mean	BPNN	120	0.504	2.860	5.925	4.401	-0.122
5	DI	mean	RF	120	0.597	2.580	5.345	4.063	0.077
5	DI	mean	SVR	120	0.445	3.027	6.271	4.484	0.101
5	DI	upper	BPNN	120	0.416	3.231	6.633	5.044	0.011
5	DI	upper	RF	120	0.446	3.146	6.457	5.042	-0.278
5	DI	upper	SVR	120	0.415	3.235	6.640	5.070	-0.137
5	SAVI	lower	BPNN	120	0.562	2.688	5.569	4.206	-0.236
5	SAVI	lower	RF	120	0.581	2.628	5.445	4.175	-0.266
5	SAVI	lower	SVR	120	0.534	2.771	5.741	4.436	-0.234
5	SAVI	mean	BPNN	120	0.425	3.206	6.580	5.007	0.000
5	SAVI	mean	RF	120	0.467	3.086	6.334	4.839	-0.166
5	SAVI	mean	SVR	120	0.381	3.325	6.825	5.293	-0.013
5	SAVI	upper	BPNN	120	0.368	3.987	8.336	6.269	-0.413
5	SAVI	upper	RF	120	0.324	4.123	8.621	6.194	-0.498
5	SAVI	upper	SVR	120	0.290	4.223	8.831	6.399	0.368
5	ZU	lower	BPNN	120	0.529	2.787	5.774	4.423	-0.309
5	ZU	lower	RF	120	0.579	2.636	5.460	4.270	-0.207
5	ZU	lower	SVR	120	0.528	2.791	5.783	4.423	-0.087
5	ZU	mean	BPNN	120	0.439	3.165	6.497	4.864	-0.047

5	ZU	mean	RF	120	0.487	3.028	6.215	4.752	-0.037
5	ZU	mean	SVR	120	0.432	3.186	6.541	5.044	-0.085
5	ZU	upper	BPNN	120	0.274	4.272	8.933	6.791	-0.292
5	ZU	upper	RF	120	0.367	3.987	8.337	6.136	-0.254
5	ZU	upper	SVR	120	0.253	4.333	9.060	6.870	0.438

Table S5. Outer-fold assignments of the 120 observations across five partitions

Repeat	SampleID	Year	PhysicalPlot	GroupFold	Repeat	SampleID	Year	PhysicalPlot	GroupFold
1	1	2022	P01	4	1	61	2023	P01	4
1	2	2022	P01	4	1	62	2023	P01	4
1	3	2022	P01	4	1	63	2023	P01	4
1	4	2022	P02	3	1	64	2023	P02	3
1	5	2022	P02	3	1	65	2023	P02	3
1	6	2022	P02	3	1	66	2023	P02	3
1	7	2022	P03	1	1	67	2023	P03	1
1	8	2022	P03	1	1	68	2023	P03	1
1	9	2022	P03	1	1	69	2023	P03	1
1	10	2022	P04	2	1	70	2023	P04	2
1	11	2022	P04	2	1	71	2023	P04	2
1	12	2022	P04	2	1	72	2023	P04	2
1	13	2022	P05	5	1	73	2023	P05	5
1	14	2022	P05	5	1	74	2023	P05	5
1	15	2022	P05	5	1	75	2023	P05	5
1	16	2022	P06	5	1	76	2023	P06	5
1	17	2022	P06	5	1	77	2023	P06	5
1	18	2022	P06	5	1	78	2023	P06	5
1	19	2022	P07	2	1	79	2023	P07	2
1	20	2022	P07	2	1	80	2023	P07	2
1	21	2022	P07	2	1	81	2023	P07	2
1	22	2022	P08	2	1	82	2023	P08	2
1	23	2022	P08	2	1	83	2023	P08	2
1	24	2022	P08	2	1	84	2023	P08	2
1	25	2022	P09	3	1	85	2023	P09	3
1	26	2022	P09	3	1	86	2023	P09	3
1	27	2022	P09	3	1	87	2023	P09	3
1	28	2022	P10	5	1	88	2023	P10	5
1	29	2022	P10	5	1	89	2023	P10	5

1	30	2022	P10	5	1	90	2023	P10	5
1	31	2022	P11	3	1	91	2023	P11	3
1	32	2022	P11	3	1	92	2023	P11	3
1	33	2022	P11	3	1	93	2023	P11	3
1	34	2022	P12	2	1	94	2023	P12	2
1	35	2022	P12	2	1	95	2023	P12	2
1	36	2022	P12	2	1	96	2023	P12	2
1	37	2022	P13	1	1	97	2023	P13	1
1	38	2022	P13	1	1	98	2023	P13	1
1	39	2022	P13	1	1	99	2023	P13	1
1	40	2022	P14	1	1	100	2023	P14	1
1	41	2022	P14	1	1	101	2023	P14	1
1	42	2022	P14	1	1	102	2023	P14	1
1	43	2022	P15	4	1	103	2023	P15	4
1	44	2022	P15	4	1	104	2023	P15	4
1	45	2022	P15	4	1	105	2023	P15	4
1	46	2022	P16	1	1	106	2023	P16	1
1	47	2022	P16	1	1	107	2023	P16	1
1	48	2022	P16	1	1	108	2023	P16	1
1	49	2022	P17	3	1	109	2023	P17	3
1	50	2022	P17	3	1	110	2023	P17	3
1	51	2022	P17	3	1	111	2023	P17	3
1	52	2022	P18	5	1	112	2023	P18	5
1	53	2022	P18	5	1	113	2023	P18	5
1	54	2022	P18	5	1	114	2023	P18	5
1	55	2022	P19	4	1	115	2023	P19	4
1	56	2022	P19	4	1	116	2023	P19	4
1	57	2022	P19	4	1	117	2023	P19	4
1	58	2022	P20	4	1	118	2023	P20	4
1	59	2022	P20	4	1	119	2023	P20	4
1	60	2022	P20	4	1	120	2023	P20	4

Table S6. Baseline and primary workflows under the original fixed five-fold and cross-year tests

Validation scheme	Target	Spectral workflow	Algorithm	R^2	RMSE	NRMSE(%)	MRE(%)
grouped_5fold	Upper-layer SPAD	Adapted baseline	BPNN	0.492	3.013	6.185	4.708
grouped_5fold	Upper-layer SPAD	DI	BPNN	0.410	3.246	6.664	4.988
grouped_5fold	Upper-layer SPAD	SAVI	BPNN	0.424	3.209	6.587	4.811
grouped_5fold	Upper-layer SPAD	ZU	BPNN	0.384	3.318	6.812	5.007
grouped_5fold	Upper-layer SPAD	Adapted baseline	SVR	0.354	3.398	6.976	5.265
grouped_5fold	Upper-layer SPAD	DI	SVR	0.350	3.408	6.995	5.340
grouped_5fold	Upper-layer SPAD	SAVI	SVR	0.345	3.421	7.023	5.132
grouped_5fold	Upper-layer SPAD	ZU	SVR	0.361	3.380	6.938	5.072
grouped_5fold	Upper-layer SPAD	Adapted baseline	RF	0.486	3.030	6.220	4.906
grouped_5fold	Upper-layer SPAD	DI	RF	0.457	3.116	6.396	4.817
grouped_5fold	Upper-layer SPAD	SAVI	RF	0.461	3.105	6.373	4.838
grouped_5fold	Upper-layer SPAD	ZU	RF	0.459	3.111	6.385	4.860
grouped_5fold	Lower-layer SPAD	Adapted baseline	BPNN	0.466	3.663	7.660	5.598
grouped_5fold	Lower-layer SPAD	DI	BPNN	0.483	3.604	7.535	5.393
grouped_5fold	Lower-layer SPAD	SAVI	BPNN	0.510	3.509	7.337	5.209
grouped_5fold	Lower-layer SPAD	ZU	BPNN	0.504	3.530	7.381	5.301
grouped_5fold	Lower-layer SPAD	Adapted baseline	SVR	0.469	3.652	7.636	5.222
grouped_5fold	Lower-layer SPAD	DI	SVR	0.466	3.665	7.663	5.164
grouped_5fold	Lower-layer SPAD	SAVI	SVR	0.460	3.683	7.700	5.242
grouped_5fold	Lower-layer SPAD	ZU	SVR	0.464	3.669	7.672	5.367
grouped_5fold	Lower-layer SPAD	Adapted baseline	RF	0.467	3.661	7.654	5.463
grouped_5fold	Lower-layer SPAD	DI	RF	0.474	3.635	7.601	5.175
grouped_5fold	Lower-layer SPAD	SAVI	RF	0.487	3.591	7.508	5.113
grouped_5fold	Lower-layer SPAD	ZU	RF	0.475	3.632	7.595	5.189
grouped_5fold	Mean SPAD	Adapted baseline	BPNN	0.629	2.473	5.123	3.917
grouped_5fold	Mean SPAD	DI	BPNN	0.617	2.515	5.209	3.881
grouped_5fold	Mean SPAD	SAVI	BPNN	0.627	2.479	5.136	3.773
grouped_5fold	Mean SPAD	ZU	BPNN	0.622	2.496	5.171	3.871
grouped_5fold	Mean SPAD	Adapted baseline	SVR	0.591	2.598	5.382	4.112

grouped_5fold	Mean SPAD	DI	SVR	0.583	2.622	5.431	4.068
grouped_5fold	Mean SPAD	SAVI	SVR	0.604	2.557	5.298	3.884
grouped_5fold	Mean SPAD	ZU	SVR	0.569	2.667	5.524	4.133
grouped_5fold	Mean SPAD	Adapted baseline	RF	0.670	2.333	4.834	3.715
grouped_5fold	Mean SPAD	DI	RF	0.636	2.450	5.076	3.810
grouped_5fold	Mean SPAD	SAVI	RF	0.636	2.450	5.076	3.894
grouped_5fold	Mean SPAD	ZU	RF	0.620	2.505	5.189	3.976
train2022_test2023	Upper-layer SPAD	Adapted baseline	BPNN	0.249	3.658	7.500	5.564
train2022_test2023	Upper-layer SPAD	DI	BPNN	0.199	3.779	7.749	5.765
train2022_test2023	Upper-layer SPAD	SAVI	BPNN	0.214	3.743	7.674	5.690
train2022_test2023	Upper-layer SPAD	ZU	BPNN	0.186	3.809	7.810	5.974
train2022_test2023	Upper-layer SPAD	Adapted baseline	SVR	0.157	3.875	7.946	5.680
train2022_test2023	Upper-layer SPAD	DI	SVR	0.191	3.796	7.784	5.591
train2022_test2023	Upper-layer SPAD	SAVI	SVR	0.205	3.764	7.719	5.571
train2022_test2023	Upper-layer SPAD	ZU	SVR	0.230	3.705	7.597	5.590
train2022_test2023	Upper-layer SPAD	Adapted baseline	RF	0.206	3.762	7.715	5.610
train2022_test2023	Upper-layer SPAD	DI	RF	0.176	3.831	7.856	5.813
train2022_test2023	Upper-layer SPAD	SAVI	RF	0.189	3.802	7.797	5.741
train2022_test2023	Upper-layer SPAD	ZU	RF	0.199	3.779	7.748	5.736
train2022_test2023	Lower-layer SPAD	Adapted baseline	BPNN	0.513	3.344	6.889	5.432
train2022_test2023	Lower-layer SPAD	DI	BPNN	0.504	3.375	6.952	5.785
train2022_test2023	Lower-layer SPAD	SAVI	BPNN	0.552	3.207	6.606	5.286
train2022_test2023	Lower-layer SPAD	ZU	BPNN	0.573	3.130	6.447	4.959
train2022_test2023	Lower-layer SPAD	Adapted baseline	SVR	0.439	3.588	7.390	5.820
train2022_test2023	Lower-layer SPAD	DI	SVR	0.538	3.257	6.710	5.253
train2022_test2023	Lower-layer SPAD	SAVI	SVR	0.555	3.194	6.579	5.095
train2022_test2023	Lower-layer SPAD	ZU	SVR	0.551	3.211	6.615	4.961
train2022_test2023	Lower-layer SPAD	Adapted baseline	RF	0.482	3.446	7.099	5.292
train2022_test2023	Lower-layer SPAD	DI	RF	0.525	3.300	6.798	5.185
train2022_test2023	Lower-layer SPAD	SAVI	RF	0.535	3.266	6.729	5.038
train2022_test2023	Lower-layer SPAD	ZU	RF	0.526	3.298	6.794	4.990
train2022_test2023	Mean SPAD	Adapted baseline	BPNN	0.674	2.213	4.549	3.607

train2022_test2023	Mean SPAD	DI	BPNN	0.629	2.359	4.849	3.866
train2022_test2023	Mean SPAD	SAVI	BPNN	0.654	2.279	4.683	3.687
train2022_test2023	Mean SPAD	ZU	BPNN	0.641	2.322	4.772	3.824
train2022_test2023	Mean SPAD	Adapted baseline	SVR	0.651	2.291	4.708	3.518
train2022_test2023	Mean SPAD	DI	SVR	0.671	2.222	4.567	3.558
train2022_test2023	Mean SPAD	SAVI	SVR	0.611	2.418	4.969	3.735
train2022_test2023	Mean SPAD	ZU	SVR	0.677	2.203	4.527	3.570
train2022_test2023	Mean SPAD	Adapted baseline	RF	0.658	2.266	4.657	3.569
train2022_test2023	Mean SPAD	DI	RF	0.648	2.301	4.728	3.743
train2022_test2023	Mean SPAD	SAVI	RF	0.655	2.276	4.678	3.655
train2022_test2023	Mean SPAD	ZU	RF	0.643	2.314	4.756	3.716
train2023_test2022	Upper-layer SPAD	Adapted baseline	BPNN	0.548	2.846	5.848	4.621
train2023_test2022	Upper-layer SPAD	DI	BPNN	0.482	3.046	6.259	4.809
train2023_test2022	Upper-layer SPAD	SAVI	BPNN	0.487	3.034	6.234	4.746
train2023_test2022	Upper-layer SPAD	ZU	BPNN	0.474	3.071	6.311	4.942
train2023_test2022	Upper-layer SPAD	Adapted baseline	SVR	0.402	3.272	6.724	5.385
train2023_test2022	Upper-layer SPAD	DI	SVR	0.383	3.324	6.831	5.400
train2023_test2022	Upper-layer SPAD	SAVI	SVR	0.386	3.316	6.814	5.358
train2023_test2022	Upper-layer SPAD	ZU	SVR	0.373	3.351	6.886	5.487
train2023_test2022	Upper-layer SPAD	Adapted baseline	RF	0.579	2.747	5.644	4.393
train2023_test2022	Upper-layer SPAD	DI	RF	0.445	3.155	6.482	4.953
train2023_test2022	Upper-layer SPAD	SAVI	RF	0.455	3.125	6.421	4.857
train2023_test2022	Upper-layer SPAD	ZU	RF	0.496	3.006	6.177	4.630
train2023_test2022	Lower-layer SPAD	Adapted baseline	BPNN	0.314	4.246	9.013	6.552
train2023_test2022	Lower-layer SPAD	DI	BPNN	0.276	4.364	9.265	6.242
train2023_test2022	Lower-layer SPAD	SAVI	BPNN	0.279	4.353	9.240	6.223
train2023_test2022	Lower-layer SPAD	ZU	BPNN	0.303	4.281	9.088	6.263
train2023_test2022	Lower-layer SPAD	Adapted baseline	SVR	0.307	4.269	9.062	6.416
train2023_test2022	Lower-layer SPAD	DI	SVR	0.184	4.631	9.830	6.850
train2023_test2022	Lower-layer SPAD	SAVI	SVR	0.187	4.623	9.813	6.846
train2023_test2022	Lower-layer SPAD	ZU	SVR	0.164	4.688	9.951	6.968
train2023_test2022	Lower-layer SPAD	Adapted baseline	RF	0.307	4.268	9.060	6.236

train2023_test2022	Lower-layer SPAD	DI	RF	0.306	4.273	9.070	6.118
train2023_test2022	Lower-layer SPAD	SAVI	RF	0.302	4.285	9.095	6.122
train2023_test2022	Lower-layer SPAD	ZU	RF	0.293	4.313	9.155	6.327
train2023_test2022	Mean SPAD	Adapted baseline	BPNN	0.566	2.769	5.782	4.521
train2023_test2022	Mean SPAD	DI	BPNN	0.577	2.735	5.711	4.350
train2023_test2022	Mean SPAD	SAVI	BPNN	0.570	2.759	5.761	4.421
train2023_test2022	Mean SPAD	ZU	BPNN	0.599	2.664	5.564	4.293
train2023_test2022	Mean SPAD	Adapted baseline	SVR	0.523	2.903	6.061	4.718
train2023_test2022	Mean SPAD	DI	SVR	0.475	3.047	6.364	4.912
train2023_test2022	Mean SPAD	SAVI	SVR	0.472	3.054	6.378	4.944
train2023_test2022	Mean SPAD	ZU	SVR	0.487	3.012	6.290	4.930
train2023_test2022	Mean SPAD	Adapted baseline	RF	0.602	2.653	5.540	4.235
train2023_test2022	Mean SPAD	DI	RF	0.601	2.657	5.548	4.206
train2023_test2022	Mean SPAD	SAVI	RF	0.611	2.624	5.479	4.097
train2023_test2022	Mean SPAD	ZU	RF	0.595	2.676	5.588	4.228

Note: Table S6 provides the complete original fixed five-fold and bidirectional cross-year results. The nine adapted-baseline models under the original fixed partition correspond to former main-text Table 10. Because the baseline and primary workflows differ in preprocessing, wavelength coverage, and candidate indices, the comparison concerns complete modeling workflows rather than a single-factor test of differentiation order or a complete reproduction of the original study.

Tble S7. Overall residual diagnostics for the 27 differentiated-spectrum models under the original fixed five-fold partition

Target	Family	Algorithm	N	Mean_observed	Mean_predicted	Bias	MAE	RMSE	R^2	Max_absolute_error	Training_mean_baseline_RMSE	Skill_vs_training_mean	Corr_pred_residual	Corr_pred_abs_residual
lower	DI	BPNN	120	47.826	47.912	0.086	2.362	3.604	0.483	16.733	5.062	0.493	0.078	-0.257
lower	DI	RF	120	47.826	47.623	-0.203	2.275	3.635	0.474	17.227	5.062	0.484	0.109	-0.275
lower	DI	SVR	120	47.826	48.322	0.496	2.210	3.665	0.466	17.110	5.062	0.476	0.101	-0.287
lower	SAVI	BPNN	120	47.826	47.921	0.095	2.265	3.509	0.510	16.771	5.062	0.519	-0.008	-0.282
lower	SAVI	RF	120	47.826	47.645	-0.181	2.252	3.591	0.487	16.814	5.062	0.497	0.116	-0.288
lower	SAVI	SVR	120	47.826	48.437	0.611	2.245	3.683	0.460	17.243	5.062	0.471	0.117	-0.289
lower	ZU	BPNN	120	47.826	47.923	0.097	2.310	3.530	0.504	16.784	5.062	0.514	0.035	-0.325
lower	ZU	RF	120	47.826	47.725	-0.101	2.281	3.632	0.475	17.258	5.062	0.485	0.122	-0.297
lower	ZU	SVR	120	47.826	48.490	0.664	2.306	3.669	0.464	17.222	5.062	0.475	0.100	-0.314
mean	DI	BPNN	120	48.271	48.331	0.060	1.787	2.515	0.617	9.855	4.123	0.628	-0.011	-0.343
mean	DI	RF	120	48.271	48.189	-0.082	1.761	2.450	0.636	10.284	4.123	0.647	0.078	-0.343
mean	DI	SVR	120	48.271	48.397	0.127	1.852	2.622	0.583	9.778	4.123	0.596	0.019	-0.397
mean	SAVI	BPNN	120	48.271	48.312	0.041	1.731	2.479	0.627	9.906	4.123	0.638	0.027	-0.319
mean	SAVI	RF	120	48.271	48.073	-0.198	1.807	2.450	0.636	9.882	4.123	0.647	0.119	-0.334
mean	SAVI	SVR	120	48.271	48.397	0.126	1.767	2.557	0.604	9.908	4.123	0.615	0.068	-0.368
mean	ZU	BPNN	120	48.271	48.321	0.050	1.780	2.496	0.622	9.905	4.123	0.633	-0.046	-0.330
mean	ZU	RF	120	48.271	48.111	-0.160	1.847	2.505	0.620	10.059	4.123	0.631	0.113	-0.337
mean	ZU	SVR	120	48.271	48.442	0.171	1.884	2.667	0.569	10.136	4.123	0.582	0.095	-0.396
upper	DI	BPNN	120	48.716	48.660	-0.056	2.347	3.246	0.410	10.101	4.309	0.432	0.078	-0.285
upper	DI	RF	120	48.716	48.600	-0.116	2.263	3.116	0.457	10.474	4.309	0.477	0.118	-0.314
upper	DI	SVR	120	48.716	48.497	-0.219	2.498	3.408	0.350	10.666	4.309	0.375	0.134	-0.286
upper	SAVI	BPNN	120	48.716	48.747	0.031	2.251	3.209	0.424	11.028	4.309	0.445	0.108	-0.294
upper	SAVI	RF	120	48.716	48.526	-0.190	2.278	3.105	0.461	10.791	4.309	0.481	0.136	-0.316
upper	SAVI	SVR	120	48.716	48.540	-0.176	2.390	3.421	0.345	11.483	4.309	0.370	0.218	-0.345
upper	ZU	BPNN	120	48.716	48.800	0.084	2.338	3.318	0.384	10.949	4.309	0.407	0.088	-0.279
upper	ZU	RF	120	48.716	48.611	-0.105	2.286	3.111	0.459	11.022	4.309	0.479	0.156	-0.295
upper	ZU	SVR	120	48.716	48.709	-0.007	2.353	3.380	0.361	11.535	4.309	0.385	0.188	-0.348

Note: Residual diagnostics in Tables S7–S10

Table S8. Original fixed five-fold predictions stratified by observation year

Algorithm	Year	N	Mean_observed	Mean_predicted	Bias	MAE	RMSE	R^2	Max_absolute_error
BPNN	2022	60	47.108	47.914	0.805	2.661	4.169	0.339	16.733
BPNN	2023	60	48.543	47.910	-0.634	2.064	2.930	0.626	8.615
RF	2022	60	47.108	47.635	0.526	2.568	4.178	0.336	17.227
RF	2023	60	48.543	47.611	-0.933	1.982	2.996	0.609	10.378
SVR	2022	60	47.108	48.324	1.216	2.606	4.298	0.297	17.110
SVR	2023	60	48.543	48.319	-0.224	1.815	2.897	0.634	9.734
BPNN	2022	60	47.108	47.923	0.815	2.575	4.082	0.366	16.771
BPNN	2023	60	48.543	47.919	-0.625	1.955	2.823	0.653	8.864
RF	2022	60	47.108	47.654	0.546	2.561	4.136	0.349	16.814
RF	2023	60	48.543	47.635	-0.908	1.942	2.946	0.622	9.656
SVR	2022	60	47.108	48.439	1.331	2.661	4.330	0.287	17.243
SVR	2023	60	48.543	48.434	-0.109	1.829	2.894	0.635	9.824
BPNN	2022	60	47.108	47.924	0.816	2.616	4.081	0.366	16.784
BPNN	2023	60	48.543	47.921	-0.622	2.003	2.874	0.640	8.611
RF	2022	60	47.108	47.730	0.622	2.541	4.137	0.349	17.258
RF	2023	60	48.543	47.720	-0.824	2.020	3.046	0.596	10.652
SVR	2022	60	47.108	48.493	1.384	2.679	4.271	0.306	17.222
SVR	2023	60	48.543	48.488	-0.055	1.934	2.947	0.622	9.659
BPNN	2022	60	47.887	48.333	0.446	1.935	2.785	0.561	9.855
BPNN	2023	60	48.655	48.330	-0.325	1.638	2.212	0.674	5.312
RF	2022	60	47.887	48.196	0.309	1.909	2.677	0.595	10.284
RF	2023	60	48.655	48.183	-0.472	1.614	2.200	0.678	5.355
SVR	2022	60	47.887	48.400	0.513	2.027	2.889	0.528	9.778
SVR	2023	60	48.655	48.395	-0.260	1.677	2.325	0.640	7.363
BPNN	2022	60	47.887	48.312	0.425	1.793	2.670	0.597	9.906
BPNN	2023	60	48.655	48.311	-0.344	1.669	2.273	0.656	6.028
RF	2022	60	47.887	48.072	0.185	1.855	2.570	0.627	9.882
RF	2023	60	48.655	48.074	-0.581	1.759	2.325	0.640	5.418
SVR	2022	60	47.887	48.397	0.511	1.903	2.772	0.565	9.908
SVR	2023	60	48.655	48.396	-0.259	1.630	2.323	0.641	7.226
BPNN	2022	60	47.887	48.322	0.435	1.892	2.729	0.579	9.905
BPNN	2023	60	48.655	48.320	-0.335	1.668	2.239	0.666	5.355
RF	2022	60	47.887	48.113	0.227	1.956	2.678	0.594	10.059
RF	2023	60	48.655	48.109	-0.546	1.739	2.318	0.642	5.530
SVR	2022	60	47.887	48.443	0.556	2.028	2.911	0.521	10.136
SVR	2023	60	48.655	48.440	-0.215	1.740	2.397	0.617	7.339
BPNN	2022	60	48.665	48.662	-0.003	1.909	2.596	0.624	7.124
BPNN	2023	60	48.767	48.659	-0.108	2.785	3.786	0.195	10.101
RF	2022	60	48.665	48.602	-0.063	1.820	2.402	0.678	6.246
RF	2023	60	48.767	48.597	-0.170	2.706	3.694	0.234	10.474
SVR	2022	60	48.665	48.499	-0.166	2.076	2.827	0.554	7.658
SVR	2023	60	48.767	48.494	-0.273	2.920	3.903	0.145	10.666
BPNN	2022	60	48.665	48.747	0.082	1.852	2.616	0.618	7.114
BPNN	2023	60	48.767	48.746	-0.021	2.651	3.708	0.228	11.028
RF	2022	60	48.665	48.521	-0.144	1.874	2.402	0.678	6.026
RF	2023	60	48.767	48.530	-0.237	2.683	3.676	0.242	10.791

SVR	2022	60	48.665	48.542	-0.123	1.918	2.765	0.573	7.963
SVR	2023	60	48.767	48.538	-0.229	2.862	3.971	0.115	11.483
BPNN	2022	60	48.665	48.801	0.136	1.916	2.753	0.577	7.657
BPNN	2023	60	48.767	48.798	0.031	2.760	3.801	0.189	10.949
RF	2022	60	48.665	48.614	-0.051	1.879	2.382	0.683	5.702
RF	2023	60	48.767	48.609	-0.159	2.693	3.698	0.232	11.022
SVR	2022	60	48.665	48.710	0.045	1.891	2.724	0.586	7.884
SVR	2023	60	48.767	48.708	-0.059	2.816	3.928	0.134	11.535

Table S9. Original fixed five-fold predictions stratified by physical plot

Target	Family	Algorithm	PhysicalPlot	N	Mean_observed	Mean_predicted	Bias	MAE	RMSE	R^2	Max_absolute_error
lower	DI	BPNN	P01	6	47.181	45.602	-1.579	2.935	3.970	0.622	8.308
lower	DI	BPNN	P02	6	44.146	44.214	0.068	2.792	4.381	0.522	8.508
lower	DI	BPNN	P03	6	50.024	48.906	-1.118	1.152	1.593	0.283	2.892
lower	DI	BPNN	P04	6	49.237	52.378	3.141	3.174	4.550	-0.539	9.871
lower	DI	BPNN	P05	6	44.252	45.044	0.792	3.298	3.781	0.406	6.387
lower	DI	BPNN	P06	6	45.880	47.002	1.122	1.315	2.288	0.874	5.241
lower	DI	BPNN	P07	6	49.370	50.295	0.925	1.082	1.301	0.848	2.285
lower	DI	BPNN	P08	6	45.940	47.303	1.363	1.479	2.315	0.893	5.146
lower	DI	BPNN	P09	6	47.672	44.335	-3.337	3.454	4.088	-0.316	6.140
lower	DI	BPNN	P10	6	51.737	50.938	-0.798	1.200	1.697	0.728	3.020
lower	DI	BPNN	P11	6	45.013	48.777	3.764	4.351	7.196	-0.183	16.733
lower	DI	BPNN	P12	6	47.775	47.604	-0.171	2.491	3.284	-0.221	6.961
lower	DI	BPNN	P13	6	44.917	48.148	3.232	3.607	5.981	-0.257	13.968
lower	DI	BPNN	P14	6	48.353	49.418	1.065	2.004	2.629	0.716	5.486
lower	DI	BPNN	P15	6	50.430	48.112	-2.318	2.318	2.393	-1.331	3.337
lower	DI	BPNN	P16	6	48.768	48.346	-0.422	1.147	1.488	0.515	3.040
lower	DI	BPNN	P17	6	48.176	47.372	-0.804	2.389	3.913	0.358	8.615
lower	DI	BPNN	P18	6	48.661	49.379	0.718	2.650	3.719	0.126	8.305
lower	DI	BPNN	P19	6	48.782	47.590	-1.192	1.678	1.786	0.070	2.649
lower	DI	BPNN	P20	6	50.203	47.471	-2.732	2.732	3.135	-0.197	5.740
lower	DI	RF	P01	6	47.181	45.650	-1.531	2.904	3.937	0.628	8.366
lower	DI	RF	P02	6	44.146	43.874	-0.272	2.818	4.151	0.571	7.638
lower	DI	RF	P03	6	50.024	49.050	-0.974	0.975	1.546	0.325	2.814
lower	DI	RF	P04	6	49.237	51.567	2.331	2.578	3.950	-0.160	8.568
lower	DI	RF	P05	6	44.252	45.037	0.785	2.824	3.590	0.464	5.702
lower	DI	RF	P06	6	45.880	46.570	0.690	1.033	1.725	0.928	4.098
lower	DI	RF	P07	6	49.370	49.518	0.148	0.273	0.401	0.986	0.892
lower	DI	RF	P08	6	45.940	47.651	1.711	1.732	2.650	0.859	5.808
lower	DI	RF	P09	6	47.672	44.094	-3.578	3.769	4.366	-0.501	6.178
lower	DI	RF	P10	6	51.737	50.573	-1.164	1.300	1.876	0.667	3.344
lower	DI	RF	P11	6	45.013	48.595	3.582	4.175	7.270	-0.207	17.227
lower	DI	RF	P12	6	47.775	48.112	0.337	2.190	3.336	-0.260	7.581
lower	DI	RF	P13	6	44.917	47.480	2.564	3.491	5.890	-0.219	13.926
lower	DI	RF	P14	6	48.353	48.746	0.393	1.472	1.994	0.836	4.131

lower	DI	RF	P15	6	50.430	47.094	-3.336	3.336	3.454	-3.855	4.324
lower	DI	RF	P16	6	48.768	48.122	-0.646	0.914	1.278	0.642	2.884
lower	DI	RF	P17	6	48.176	46.860	-1.316	2.216	4.344	0.209	10.378
lower	DI	RF	P18	6	48.661	49.092	0.431	3.134	4.236	-0.134	8.781
lower	DI	RF	P19	6	48.782	47.366	-1.416	1.565	2.034	-0.205	4.262
lower	DI	RF	P20	6	50.203	47.403	-2.800	2.800	3.285	-0.314	5.644
lower	DI	SVR	P01	6	47.181	46.240	-0.941	2.937	3.754	0.662	7.723
lower	DI	SVR	P02	6	44.146	43.998	-0.148	2.575	3.772	0.645	6.892
lower	DI	SVR	P03	6	50.024	49.494	-0.530	0.972	1.278	0.539	2.194
lower	DI	SVR	P04	6	49.237	52.035	2.798	2.860	4.439	-0.465	9.957
lower	DI	SVR	P05	6	44.252	46.072	1.820	3.143	4.347	0.215	9.734
lower	DI	SVR	P06	6	45.880	48.149	2.269	2.269	3.759	0.659	7.928
lower	DI	SVR	P07	6	49.370	50.242	0.872	0.872	0.965	0.916	1.367
lower	DI	SVR	P08	6	45.940	47.506	1.566	1.714	2.654	0.859	5.844
lower	DI	SVR	P09	6	47.672	44.112	-3.560	3.739	4.460	-0.566	6.314
lower	DI	SVR	P10	6	51.737	51.041	-0.695	1.114	1.519	0.782	2.667
lower	DI	SVR	P11	6	45.013	49.104	4.091	4.341	7.313	-0.222	17.110
lower	DI	SVR	P12	6	47.775	48.735	0.960	1.867	3.389	-0.299	8.044
lower	DI	SVR	P13	6	44.917	48.249	3.332	3.422	6.245	-0.370	14.828
lower	DI	SVR	P14	6	48.353	49.768	1.415	1.712	2.540	0.735	5.414
lower	DI	SVR	P15	6	50.430	49.020	-1.410	1.410	1.481	0.108	1.920
lower	DI	SVR	P16	6	48.768	48.515	-0.254	0.847	1.071	0.749	2.209
lower	DI	SVR	P17	6	48.176	47.572	-0.603	2.250	3.885	0.367	8.524
lower	DI	SVR	P18	6	48.661	50.032	1.372	2.560	3.800	0.088	8.789
lower	DI	SVR	P19	6	48.782	48.925	0.143	1.016	1.366	0.456	2.872
lower	DI	SVR	P20	6	50.203	47.620	-2.583	2.583	3.263	-0.297	6.482

Table S10. Original fixed five-fold predictions stratified by measured-SPAD quartiles

Target	Family	Algorithm	Observed_quartile	Lower	Upper	N	Mean_observed	Mean_predicted	Bias	MAE	RMSE	R²	Max_absolute_error
lower	DI	BPNN	1	31.760	45.773	30	40.960	44.359	3.400	4.555	5.929	-0.932	16.733
lower	DI	BPNN	2	45.773	48.430	29	47.287	47.047	-0.241	1.161	1.945	-4.758	8.308
lower	DI	BPNN	3	48.430	51.320	30	49.850	48.517	-1.333	2.016	2.601	-6.452	6.439
lower	DI	BPNN	4	51.320	55.740	31	53.016	51.573	-1.443	1.700	2.482	-3.411	8.615
lower	DI	RF	1	31.760	45.773	30	40.960	43.894	2.935	4.318	5.801	-0.850	17.227
lower	DI	RF	2	45.773	48.430	29	47.287	47.006	-0.281	0.831	1.750	-3.664	8.366
lower	DI	RF	3	48.430	51.320	30	49.850	48.097	-1.753	2.160	2.851	-7.955	6.472
lower	DI	RF	4	51.320	55.740	31	53.016	51.349	-1.667	1.761	2.802	-4.624	10.378
lower	DI	SVR	1	31.760	45.773	30	40.960	44.662	3.702	4.911	6.375	-1.233	17.110
lower	DI	SVR	2	45.773	48.430	29	47.287	47.310	0.023	0.951	1.714	-3.473	7.723
lower	DI	SVR	3	48.430	51.320	30	49.850	49.014	-0.836	1.677	2.348	-5.075	6.482
lower	DI	SVR	4	51.320	55.740	31	53.016	52.139	-0.877	1.291	2.142	-2.286	8.524
lower	SAVI	BPNN	1	31.760	45.773	30	40.960	44.550	3.590	4.595	5.890	-0.906	16.771
lower	SAVI	BPNN	2	45.773	48.430	29	47.287	46.958	-0.329	0.920	1.454	-2.221	6.253
lower	SAVI	BPNN	3	48.430	51.320	30	49.850	48.475	-1.375	1.979	2.560	-6.217	6.046
lower	SAVI	BPNN	4	51.320	55.740	31	53.016	51.547	-1.468	1.545	2.404	-3.140	8.563
lower	SAVI	RF	1	31.760	45.773	30	40.960	43.756	2.797	4.367	5.765	-0.827	16.814
lower	SAVI	RF	2	45.773	48.430	29	47.287	47.133	-0.154	0.758	1.842	-4.168	9.297
lower	SAVI	RF	3	48.430	51.320	30	49.850	48.230	-1.620	2.032	2.676	-6.889	5.794
lower	SAVI	RF	4	51.320	55.740	31	53.016	51.320	-1.696	1.815	2.766	-4.479	9.656
lower	SAVI	SVR	1	31.760	45.773	30	40.960	44.774	3.815	5.094	6.418	-1.264	17.243
lower	SAVI	SVR	2	45.773	48.430	29	47.287	47.387	0.100	0.953	1.733	-3.573	7.613
lower	SAVI	SVR	3	48.430	51.320	30	49.850	49.068	-0.783	1.658	2.415	-5.425	6.684
lower	SAVI	SVR	4	51.320	55.740	31	53.016	52.353	-0.663	1.266	2.045	-1.995	7.944
lower	ZU	BPNN	1	31.760	45.773	30	40.960	44.484	3.525	4.818	5.941	-0.940	16.784
lower	ZU	BPNN	2	45.773	48.430	29	47.287	46.907	-0.381	0.961	1.673	-3.264	7.541
lower	ZU	BPNN	3	48.430	51.320	30	49.850	48.584	-1.266	1.836	2.340	-5.030	5.743
lower	ZU	BPNN	4	51.320	55.740	31	53.016	51.561	-1.455	1.603	2.482	-3.413	8.611
lower	ZU	RF	1	31.760	45.773	30	40.960	44.000	3.040	4.465	5.823	-0.863	17.258
lower	ZU	RF	2	45.773	48.430	29	47.287	46.916	-0.371	0.752	1.817	-4.029	9.278
lower	ZU	RF	3	48.430	51.320	30	49.850	48.404	-1.447	2.038	2.621	-6.569	5.498

lower	ZU	RF	4	51.320	55.740	31	53.016	51.429	-1.586	1.832	2.920	-5.104	10.652
lower	ZU	SVR	1	31.760	45.773	30	40.960	44.992	4.032	5.282	6.408	-1.257	17.222
lower	ZU	SVR	2	45.773	48.430	29	47.287	47.308	0.021	0.877	1.711	-3.459	7.664
lower	ZU	SVR	3	48.430	51.320	30	49.850	49.188	-0.662	1.747	2.294	-4.799	7.338
lower	ZU	SVR	4	51.320	55.740	31	53.016	52.307	-0.708	1.305	2.130	-2.248	7.953

Table S11. Summary of five validation repetitions for NDI

Spectral processing	Target	Algorithm	NDI_R^2_mean	NDI_R^2_SD	NDI_R^2_min	NDI_R^2_max	NDI_RMSE_mean	NDI_RMSE_SD	DI_R^2_mean	DI_RMSE_mean	Mean_delta_RMSE	Repeats_NDI_better
Fractional-order differentiated spectra	lower	BPNN	0.333	0.088	0.225	0.403	4.087	0.268	0.430	3.778	0.309	0
	lower	RF	0.400	0.081	0.304	0.478	3.878	0.261	0.407	3.855	0.022	3
	lower	SVR	0.284	0.085	0.140	0.368	4.238	0.246	0.385	3.921	0.317	0
	mean	BPNN	0.538	0.075	0.418	0.600	2.754	0.220	0.575	2.637	0.117	0
	mean	RF	0.593	0.057	0.493	0.633	2.588	0.174	0.589	2.598	-0.010	2
	mean	SVR	0.488	0.081	0.348	0.556	2.898	0.222	0.540	2.747	0.151	1
	upper	BPNN	0.362	0.043	0.321	0.426	3.376	0.116	0.399	3.275	0.101	1
	upper	RF	0.395	0.034	0.346	0.425	3.288	0.093	0.435	3.177	0.111	0
	upper	SVR	0.305	0.060	0.260	0.410	3.522	0.157	0.363	3.372	0.150	1
Undifferentiated smoothed spectra	lower	BPNN	0.273	0.117	0.076	0.369	4.263	0.334	0.364	3.988	0.275	1
	lower	RF	0.330	0.139	0.098	0.443	4.089	0.410	0.404	3.861	0.227	0
	lower	SVR	0.196	0.128	-0.028	0.282	4.484	0.342	0.331	4.098	0.386	1
	mean	BPNN	0.362	0.210	-0.002	0.539	3.214	0.498	0.527	2.791	0.423	1
	mean	RF	0.506	0.208	0.134	0.609	2.814	0.541	0.579	2.635	0.179	3

	mean	SVR	0.330	0.202	-0.017	0.494	3.297	0.469	0.530	2.783	0.514	0
	upper	BPNN	0.250	0.208	-0.110	0.384	3.637	0.475	0.326	3.461	0.177	1
	upper	RF	0.382	0.057	0.295	0.425	3.321	0.152	0.395	3.285	0.036	0
	upper	SVR	0.227	0.081	0.103	0.296	3.713	0.193	0.331	3.452	0.261	0

Table S12. Differences between DI and NDI metrics under identical partitions

Repeat	Scheme	Spectral processing	Target	Algorithm	DI_R²	NDI_R²	Delta_R²	DI_RMSE	NDI_RMSE	Delta_RMSE	DI_NRMSE	NDI_NRMSE
1	grouped_5fold	Fractional-order differentiated spectra	lower	BPNN	0.483	0.386	-0.097	3.604	3.927	0.323	7.535	8.211
1	grouped_5fold		lower	RF	0.474	0.478	0.003	3.635	3.624	-0.012	7.601	7.577
1	grouped_5fold		lower	SVR	0.466	0.368	-0.098	3.665	3.987	0.321	7.663	8.336
1	grouped_5fold		mean	BPNN	0.617	0.600	-0.017	2.515	2.568	0.054	5.209	5.321
1	grouped_5fold		mean	RF	0.636	0.611	-0.025	2.450	2.532	0.082	5.076	5.246
1	grouped_5fold		mean	SVR	0.583	0.500	-0.084	2.622	2.873	0.251	5.431	5.952
1	grouped_5fold		upper	BPNN	0.410	0.380	-0.030	3.246	3.329	0.083	6.664	6.833
1	grouped_5fold		upper	RF	0.457	0.373	-0.084	3.116	3.348	0.232	6.396	6.873
1	grouped_5fold		upper	SVR	0.350	0.260	-0.091	3.408	3.638	0.230	6.995	7.467
1	grouped_5fold	Undifferentiated smoothed spectra	lower	BPNN	0.451	0.355	-0.096	3.715	4.025	0.311	7.767	8.417
1	grouped_5fold		lower	RF	0.496	0.443	-0.053	3.558	3.742	0.183	7.440	7.824
1	grouped_5fold		lower	SVR	0.399	0.222	-0.177	3.886	4.423	0.537	8.126	9.248
1	grouped_5fold		mean	BPNN	0.493	0.419	-0.074	2.891	3.096	0.205	5.989	6.414

1	grouped_5fold		mean	RF	0.580	0.599	0.019	2.633	2.572	-0.061	5.456	5.329
1	grouped_5fold		mean	SVR	0.559	0.437	-0.122	2.696	3.048	0.352	5.586	6.314
1	grouped_5fold		upper	BPNN	0.323	0.354	0.031	3.478	3.397	-0.081	7.140	6.973
1	grouped_5fold		upper	RF	0.420	0.416	-0.004	3.221	3.231	0.010	6.611	6.632
1	grouped_5fold		upper	SVR	0.316	0.248	-0.069	3.496	3.667	0.171	7.175	7.527
1	train2022_test2023		lower	BPNN	0.504	0.466	-0.037	3.375	3.499	0.124	6.952	7.208
1	train2022_test2023		lower	RF	0.525	0.502	-0.024	3.300	3.382	0.081	6.798	6.966
1	train2022_test2023		lower	SVR	0.538	0.383	-0.154	3.257	3.761	0.504	6.710	7.748
1	train2022_test2023		mean	BPNN	0.629	0.580	-0.050	2.359	2.512	0.153	4.849	5.164
1	train2022_test2023	Fractional-order differentiated spectra	mean	RF	0.648	0.646	-0.001	2.301	2.305	0.004	4.728	4.737
1	train2022_test2023		mean	SVR	0.671	0.552	-0.119	2.222	2.593	0.371	4.567	5.329
1	train2022_test2023		upper	BPNN	0.199	0.206	0.008	3.779	3.761	-0.018	7.749	7.711
1	train2022_test2023		upper	RF	0.176	0.184	0.007	3.831	3.814	-0.017	7.856	7.821
1	train2022_test2023		upper	SVR	0.191	0.150	-0.042	3.796	3.892	0.096	7.784	7.982
1	train2022_test2023		lower	BPNN	0.502	0.422	-0.080	3.380	3.640	0.261	6.962	7.499
1	train2022_test2023		lower	RF	0.508	0.518	0.009	3.359	3.327	-0.032	6.920	6.853
1	train2022_test2023	Undifferentiated smoothed spectra	lower	SVR	0.476	0.401	-0.075	3.469	3.707	0.238	7.146	7.637
1	train2022_test2023		mean	BPNN	0.624	0.511	-0.114	2.376	2.711	0.335	4.883	5.573
1	train2022_test2023		mean	RF	0.651	0.603	-0.048	2.289	2.441	0.153	4.704	5.017

1	train2022_test2023		mean	SVR	0.609	0.482	-0.127	2.424	2.790	0.366	4.983	5.734
1	train2022_test2023		upper	BPNN	0.174	0.142	-0.032	3.836	3.910	0.074	7.866	8.018
1	train2022_test2023		upper	RF	0.179	0.156	-0.023	3.825	3.878	0.053	7.843	7.951
1	train2022_test2023		upper	SVR	0.170	0.134	-0.036	3.845	3.928	0.084	7.884	8.055
1	train2023_test2022	Fractional-order differentiated spectra	lower	BPNN	0.276	0.352	0.076	4.364	4.128	-0.236	9.265	8.764
1	train2023_test2022		lower	RF	0.306	0.350	0.044	4.273	4.135	-0.138	9.070	8.777
1	train2023_test2022		lower	SVR	0.184	0.198	0.013	4.631	4.593	-0.038	9.830	9.749
1	train2023_test2022		mean	BPNN	0.577	0.601	0.024	2.735	2.655	-0.080	5.711	5.543
1	train2023_test2022		mean	RF	0.601	0.614	0.014	2.657	2.611	-0.046	5.548	5.452
1	train2023_test2022		mean	SVR	0.475	0.447	-0.027	3.047	3.125	0.078	6.364	6.527
1	train2023_test2022		upper	BPNN	0.482	0.446	-0.036	3.046	3.150	0.104	6.259	6.473
1	train2023_test2022	Undifferentiated smoothed spectra	upper	RF	0.445	0.463	0.018	3.155	3.102	-0.052	6.482	6.375
1	train2023_test2022		upper	SVR	0.383	0.324	-0.060	3.324	3.481	0.157	6.831	7.153
1	train2023_test2022		lower	BPNN	0.269	0.194	-0.075	4.383	4.602	0.219	9.304	9.769
1	train2023_test2022		lower	RF	0.286	0.306	0.019	4.332	4.273	-0.059	9.196	9.070
1	train2023_test2022		lower	SVR	0.152	0.103	-0.049	4.721	4.856	0.135	10.021	10.308
1	train2023_test2022		mean	BPNN	0.518	0.496	-0.023	2.919	2.986	0.068	6.095	6.236
1	train2023_test2022		mean	RF	0.558	0.609	0.051	2.797	2.630	-0.167	5.840	5.492

1	train2023_test2022	mean	SVR	0.515	0.341	-0.174	2.929	3.413	0.484	6.116	7.128
1	train2023_test2022	upper	BPNN	0.492	0.356	-0.135	3.018	3.397	0.378	6.203	6.980
1	train2023_test2022	upper	RF	0.507	0.494	-0.013	2.973	3.011	0.038	6.109	6.187
1	train2023_test2022	upper	SVR	0.385	0.238	-0.147	3.321	3.696	0.375	6.823	7.594

Table S13. Out-of-fold comparison of direct mean-SPAD prediction and equal-weight averaging of layer-specific predictions

Input feature set	Algorithm	Prediction strategy	N	R^2	RMSE	NRMSE (%)	MRE (%)
DI	RF	Direct mean-SPAD prediction	120	0.636	2.450	5.076	3.810
DI	RF	Equal-weight mean of layer-specific predictions	120	0.634	2.457	5.090	3.822
DI	SVR	Direct mean-SPAD prediction	120	0.583	2.622	5.431	4.068
DI	SVR	Equal-weight mean of layer-specific predictions	120	0.569	2.666	5.523	4.037
DI	BPNN	Direct mean-SPAD prediction	120	0.617	2.515	5.209	3.881
DI	BPNN	Equal-weight mean of layer-specific predictions	120	0.607	2.546	5.274	3.928
SAVI	RF	Direct mean-SPAD prediction	120	0.636	2.450	5.076	3.894
SAVI	RF	Equal-weight mean of layer-specific predictions	120	0.639	2.441	5.057	3.831
SAVI	SVR	Direct mean-SPAD prediction	120	0.604	2.557	5.298	3.884
SAVI	SVR	Equal-weight mean of layer-specific predictions	120	0.552	2.720	5.634	4.129
SAVI	BPNN	Direct mean-SPAD prediction	120	0.627	2.479	5.136	3.773
SAVI	BPNN	Equal-weight mean of layer-specific predictions	120	0.619	2.507	5.194	3.879
ZU	RF	Direct mean-SPAD prediction	120	0.620	2.505	5.189	3.976
ZU	RF	Equal-weight mean of layer-specific predictions	120	0.646	2.418	5.009	3.775
ZU	SVR	Direct mean-SPAD prediction	120	0.569	2.667	5.524	4.133
ZU	SVR	Equal-weight mean of layer-specific predictions	120	0.567	2.673	5.538	4.094
ZU	BPNN	Direct mean-SPAD prediction	120	0.622	2.496	5.171	3.871
ZU	BPNN	Equal-weight mean of layer-specific predictions	120	0.609	2.539	5.260	3.946

Note: All comparisons use the same evaluation target and observations. Differences are descriptive and do not establish statistical significance. RMSE is in SPAD units; NRMSE and MRE are percentages. The table covers only the original fixed grouped five-fold partition, without repeated grouped or cross-year comparisons of these strategies.

Table S14. Selected observations and within-year SPAD grouping boundaries for Figure S14

Sample ID	Year	Physical plot	Within-year mean-SPAD group	Mean SPAD	Upper SPAD	Lower SPAD	Within-year group interval
31	2022	P11	Low	44.220	45.700	42.740	≤47.073300
4	2022	P02	Medium	48.715	49.200	48.230	>47.073300 and ≤50.018333
50	2022	P17	High	51.460	50.130	52.790	>50.018333
109	2023	P17	Low	46.345	39.900	52.790	≤47.756667
107	2023	P16	Medium	49.440	50.320	48.560	>47.756667 and ≤50.103333
84	2023	P08	High	51.610	56.900	46.320	>50.103333

Note: Groups were defined using the one-third and two-thirds quantiles of mean SPAD among the 60 observations in each year. The observation closest to each group median was selected; ties were resolved by sample ID order. Low, medium, and high denote within-year mean-SPAD groups, not canopy positions. Boundaries are displayed to six decimal places, whereas selection used unrounded values. SPAD values are shown to three decimal places. Figure S14 and Table S14 belong to separate supplementary figure and table numbering series.
